# Lightweight Aggregate Concrete with Regard to Bridge Structures—State of the Art

**DOI:** 10.3390/ma18163874

**Published:** 2025-08-19

**Authors:** Marcin Piechaczek, Krzysztof Adam Ostrowski, Kazimierz Furtak

**Affiliations:** 1 CUT Doctoral School, Faculty of Civil Engineering, Cracow University of Technology, ul. Warszawska 24, 31-155 Cracow, Poland; 2 Faculty of Civil Engineering, Cracow University of Technology, ul. Warszawska 24, 31-155 Cracow, Poland; krzysztof.ostrowski.1@pk.edu.pl (K.A.O.); kfurtak@pk.edu.pl (K.F.)

**Keywords:** concrete, bridge structures, lightweight aggregate, lightweight structural concrete

## Abstract

The article presents a recognition of the current state of the art in the field of bridge structures made using concrete on lightweight aggregate. The article aims to show the reader why aggregate with low mechanical parameters and high absorption can be used in demanding bridge constructions. Divided into two parts, the first presents the history of both topics and compiles the parameters of currently used lightweight aggregates by considering the guidelines applicable in the EU, China, and America concerning bridge construction. The literature review conducted highlighted both the advantages and disadvantages of using lightweight aggregates, presented the knowledge accumulated to date in this area, and identified important research gaps regarding lightweight aggregates. The second part discusses existing or planned bridge structures, taking into account their shapes and material properties. In summary, the challenges involved in the development of lightweight aggregate for bridge structures. The results obtained from the analysis will provide a basis for further research into the development of original lightweight aggregate for bridge structures.

## 1. Introduction

Lightweight aggregates and bridge structures seem to be completely unrelated. Lightweight aggregates are associated with porous, lightweight, and brittle materials, and are often used in fields like horticulture. In construction, lightweight aggregates are employed as thermal insulation materials, or as aggregates for producing lightweight concrete. However, modern bridges are characterized by reduced mass, allowing structures with longer spans and less massive construction, increasing the efficiency and economy of their design and construction. Therefore, the use of lightweight aggregates as construction aggregates in lightweight concrete for bridge structures seems doubtful.

### 1.1. A Brief History of Lightweight Bridges

The definition of a “lightweight bridge” is relatively broad, allowing a wide range of structural types to be classified under this term. From an engineering perspective, lightweight bridges are structures characterized by significantly reduced self-weight compared to conventional designs. It is worth noting that the self-weight of a bridge can account for up to 90% of the total design load. This highlights the need to employ materials with low density and high mechanical strength, enabling the construction of longer spans without the use of intermediate supports. Such an approach reduces the consumption of raw materials.

The history of lightweight bridge structures dates to the same period as the emergence of massive constructions. These types of bridges were initially constructed over ravines where the placement of intermediate support was not feasible. The load-bearing capacity of such structures relied on ropes stretched across the chasm, with loosely bound planks serving as the deck. Despite their advantages, these constructions exhibited numerous drawbacks. The low mass of the bridge made it susceptible to oscillations induced by wind, leading to limited usability under adverse weather conditions and reduced durability. Nevertheless, these structures were employed not only for communication purposes but also for defensive applications, such as drawbridges. Another example of lightweight bridge structures includes pontoon bridges, primarily used for military purposes [1]. These bridges consisted of a series of boats tied together with ropes, upon which a deck made of wooden planks was laid. Such solutions were intended for temporary use and were not designed as permanent structures. Consequently, it is worth emphasizing that lightweight bridges were inherently temporary solutions, not meant for long-term usage. These structures were vulnerable to atmospheric conditions and, due to the use of lightweight plant-based materials, were prone to biological degradation. This historical context underlines the transitional and often provisional nature of lightweight bridge construction. However, the real breakthrough came with the widespread use of structural steel in bridge construction. Steel enabled a significant reduction in the weight of constructions, and at the same time lowered construction costs. One of the first significant steel-based bridge structures was the Eads Bridge in Illinois, USA [2]. Even earlier than this, steel in the form of chains and cables was employed in lightweight bridge structures with wooden decking. Initially, these were mainly drawbridges used for defense in castles and fortresses. With the increased use of steel, the possibility of constructing lighter bridge structures emerged.

The first project of its kind, serving as a prototype for both future suspension and cable-stayed constructions, was developed in France in the early 17th century [3]. Similar designs were also constructed until the early 19th century, when steel began to be used for their realization [4]. This marked the birth of modern lightweight bridge structures. In 1801, James Finley was granted a patent for the first suspension bridge, which was completed in 1801 as a crossing over Jacob’s Creek [5]. The initial designs were based on the experience of contemporary builders, who employed traditional methods of structural analysis and dimensioning [6]. However, structures like Kings’s Meadows and Dryburgh Abbey in Scotland, based on the mentioned publication, were destroyed shortly after use.

The problem of inadequate structural strength in lightweight structures arose primarily from a lack of understanding of their structural behavior. Similarly to earlier lightweight constructions, these structures were characterized by low resistance to environmental influences. The first publication concerning the design of suspension structures was published in 1811 [7]. This marked a pivotal moment, emphasizing the need to develop design guidelines to facilitate the construction of such structures. The introduction of these guidelines aimed to improve both the durability and reliability of lightweight structures, to meet the challenges posed by environmental conditions and to ensure their structural integrity. French engineer and mathematician Claude-Louis Navier drew inspiration from English designs to publish “Rapport à Monsieur Becquey et Mémoire sur les PontsSuspendus” [8]. This work consolidated existing knowledge on the design and construction of suspension bridges, serving as a key reference for the field. Navier’s guidelines, rooted in elasticity theory, lasted until the early 20th century [9]. Then, based on theoretical analyses proposed by Ritter and Melan [10], an innovative theory of deflection was developed, more accurately reflecting the behavior of suspension bridges. However, it did not account for aerodynamic forces, which led to the collapse of the Tacoma Narrows Bridge in 1940 [11]. This incident introduced dynamic analysis into the design process, with wind and earthquake loads being considered. This allowed for the design of even lighter bridge structures with significantly larger spans. As a civilization, drawing on the experience of our ancestors and by accessing international technologies, we are capable of constructing diverse bridge structures [12].

Advanced materials like fiber-reinforced polymers allow for a further reduction in the mass of structures, while at the same time increasing their load-bearing capacity [13,14,15]. Examples of such structures can be found in China, the United Kingdom, and Poland. Advanced 3D printing technology, which utilizes fine aggregate concrete, permits the creation of lightweight bridge structures with intriguing geometry. An example is the Wisdom Bay Pond Bridge in Shanghai [16]. With the use of novel materials and advanced technologies, our capabilities in bridge construction continue to grow, in turn opening new horizons in the field of bridge engineering.

One of the effective methods for reducing the self-weight of bridge structures is the use of lightweight aggregates as filler material in the production of structural lightweight concrete. This approach enables the creation of concrete with reduced density while maintaining the mechanical performance required for load-bearing elements. Lightweight concrete not only meets strength requirements but also exhibits improved seismic resistance due to its lower dynamic mass, making it particularly suitable for earthquake-prone regions. Importantly, the use of lightweight aggregates—often derived from industrial by-products or recycled materials—also reduces the demand for natural aggregates, thereby supporting sustainable development and the preservation of environmental resources.

This study aims to introduce the reader to the idea of using lightweight aggregates in bridge engineering. It presents the history of constructing bridge structures, highlighting the advantages of lightweight aggregates, an analysis of physical and chemical research conducted on lightweight aggregates and lightweight aggregate concrete, as well as a detailed description of bridge structures built using lightweight aggregate concrete technology.

### 1.2. A Brief History of Lightweight Aggregates

The definition of lightweight aggregate appears to be more specific. According to the current European standards [17,18,19], lightweight aggregate for concrete is defined as “mineral-origin aggregate with a grain density in dry state not exceeding 2000 kg/m^3^, or bulk density in loose state not exceeding 1200 kg/m^3^.” Additionally, lightweight aggregates are categorized into four separate categories based on their origin: natural, artificial, waste, and recycled. On the other hand, American standards [20,21] provide upper limits for bulk densities: 1120 kg/m^3^ for fine lightweight aggregates, 880 kg/m^3^ for coarse lightweight aggregates, and 1040 kg/m^3^ for a combination of fine and coarse lightweight aggregates. Furthermore, the aforementioned ASTM standards clearly define the classification of lightweight aggregates obtained through the mechanical processing of natural materials, such as pumice, and those produced through expansion, granulation, or sintering. The American guidelines also present a division of lightweight concretes into “all-lightweight concrete” with coarse and fine lightweight aggregates, and “sand-lightweight concrete” with coarse lightweight aggregates and sand [22]. Chinese guidelines on lightweight aggregates in the GB/T17431.2-2010 standard [23] are based on assumptions contained in the current European standards [17,18,19].

**Figure 1:**
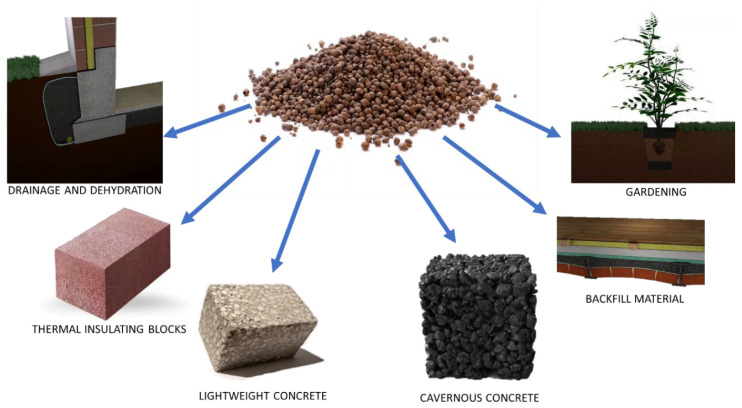
The scope of lightweight aggregate applications.

The historical beginnings of using lightweight aggregates can be traced back to ancient Rome [24]. Roman builders utilized natural pumice, which when combined with volcanic ash or lime, allowed for the production of cementitious mortars. Mixtures like these were used in the construction of structures like the Pantheon [25]. In later centuries, due to a lack of natural materials, concrete structures utilizing lightweight aggregate faded away. The beginning of the 20th century, with the emergence of the first commercial expanded aggregate plant, changed this [26]. Initially, a concrete mixture based on patented lightweight aggregate, Haydite (U.S. Patent No. 1,255,878) [27], was used as fill for naval ship hulls in the United States. After the war, in the 1920s, lightweight aggregate was used in bridge construction [28,29]. In later years, it began to also be used in civil construction [30]. Currently, lightweight aggregates have applications in various branches of construction, with its distinct properties being utilized (see Figure 1). Based on report [31] it is forecasted that the global market for lightweight aggregates used in concrete production will grow and reach a value of approximately $6.1 billion by 2030.

Residential construction, utilizing the low density and thermal insulation of lightweight aggregates, employs them to relieve beam ceilings [32]. Lightweight aggregate is also used to produce the thermal insulation bricks [33] that meet current thermal insulation guidelines for single- and triple-layer partitions [34]. Due to its water permeability, lightweight aggregate is also applied in drainage systems [35]. In road construction, lightweight aggregates are used to reinforce and shape road embankments. Efforts are also underway to employ lightweight aggregates in the creation of water-permeable surfaces. Lightweight aggregates are also used to produce the lightweight structural concretes used in the construction of office buildings, industrial structures, and bridges. The reason for using lightweight structural concrete is their low mass-to-strength ratio and their ability to absorb seismic and post-seismic shocks.

The fundamental advantage of lightweight aggregates is their porous structure, which helps reduce the weight of structures. Reducing self-weight while at the same time maintaining relatively high strength parameters enables the repair and relief of existing structures. Due to their structure, lightweight aggregates are also used in thermal and acoustic insulation [36]. The thermal insulation aspect has been utilized in the production of ceramic bricks and lightweight fills for quiet floors. Reducing the weight of structures also positively impacts their seismic resistance. This is because the mass of the structure influences the frequency and form of its natural vibrations. However, in the case of concrete with lightweight aggregate, attention must also be paid to their compressive strength and the modulus of elasticity of the lightweight aggregate itself. In study [37], it was noted that while lightweight aggregates reduce seismic loading, the aggregate may shear or crush when subjected to a load. Low mechanical strength is one of the key drawbacks of lightweight aggregates. According to the EN 13055 standard [17], the crushing strength of lightweight aggregates ranges from 0.5 to 10 MPa. This means that in concrete mixes that use lightweight aggregates, the mortar plays a primary role in the strength of the concrete mix. This is not the case in mixes that use normal aggregates. To compensate for the low strength of lightweight aggregates, normal aggregates and sand are also added to the mix. Due to the porous structure of lightweight aggregates, and the fact that their modulus of elasticity is similar to that of mortar, stresses in the concrete are evenly distributed. The penetration of mortar into the aggregate structure strengthens the contact zone between the grains and mortar, where damage propagation occurs. Therefore, the destruction of concrete propagates inside the porous lightweight aggregate. Furthermore, due to their high porosity, lightweight aggregates absorb more water when compared to natural aggregates. Improperly prepared lightweight aggregates can absorb water from the cement paste. This significantly affects the workability and pumpability of concrete mixes containing lightweight aggregates. Additionally, reducing the water-to-cement ratio (w/c) can significantly affect the concrete. A decrease in the amount of water in the cement mixture can result in insufficient moistening of cement particles, thereby limiting the hydration process. This is one of the reasons why contractors and designers have been reluctant to use lightweight aggregates. However, pre-wetting the aggregate eliminates workability issues and additionally densifies the paste structure in the contact zone with the aggregate grain. The topic of sealing lightweight concrete in the contact zone is described in detail in study [30].

Currently, depending on the world region and available deposits, the production of lightweight aggregates looks different. For example, analyzing the American market reveals many manufacturers of lightweight aggregates, the composition of which depends on the available mineral deposits. For instance, Stalite Lightweight AGGREGATE [38] is produced using locally available volcanic ash deposits. The mineral composition and conditions of material formation contributed to the possibility of obtaining lightweight aggregates with a relatively high compressive strength-to-weight ratio [39]. This is why this aggregate has been used in the construction of Norwegian lightweight bridge structures with cantilever or floating designs. Moreover, in the United States, lightweight aggregates like Utelite [40], Solite [41], and ARCOSA Lightweight [42] are produced. In the Old Continent region, the prevalence of LECA lightweight aggregate is noticeable. This aggregate, originating from Denmark, is currently produced in almost every country in Europe. Competing with this type of aggregate is the lightweight aggregate Lytag. Importantly, LECA Ceramsite represents clay-based lightweight aggregate, while Lytag is produced from fly ash from coal-fired power plants. Other aggregates, produced more for local markets, include Laterlite [43], produced in Italy, and Mullite [44], produced in France. In Poland, the leading producers of lightweight aggregates are manufacturing plants in Gniew, Mszczonów (LECA Ceramsite), and Białystok (Certyd [45,46]). In Asia, various productions of lightweight aggregates exist, such as Nodullar [47], produced in India, Mezalite, produced in Japan, and Bazelite, produced in the People’s Republic of China.

## 2. The Aim and Scope of This Study

The aim of this paper is to present the specifics of designing and constructing bridge structures that partially or entirely use lightweight aggregate concrete. To achieve this, a comprehensive literature review of works related to bridge construction using lightweight aggregate concrete was conducted. This review serves as an introduction to further research in the field of the lightweight aggregates used in bridge engineering. The acquired knowledge will contribute to the development of proprietary waste lightweight aggregates.

The first part of the study provides a preliminary literature review based on keywords from popular library catalogs. This review was carried out using collections from three leading bibliometric catalogs: Scopus, LENS, and WorldCat. Based on the results obtained from this analysis, areas requiring further research were identified.

In Section 4, the physicochemical characteristics of selected lightweight aggregates, and the concrete produced from them, are described based on selected scientific publications. This description presents a range of benefits of using lightweight aggregates, but also highlights issues requiring further analysis. The second part of Section 4 examines the current guidelines for the design and construction of concrete structures using lightweight aggregates, with a specific focus on bridge structures. Guidelines applicable in the United States, Europe, and the People’s Republic of China are compared.

Section 5 presents, based on the literature review, the implementations of utilizing lightweight aggregate in the construction of bridge structures. This description is divided into subsections describing bridge structures from different regions of the world. Due to the number of such structures, the decision was made to describe the most representative ones. Other structures not included in the paper are summarized in tables. The final subsection refers to examples of using lightweight aggregate concrete for building tunnels, and the concept of using this material for innovative underwater crossings.

The last part of the paper, Section 6, provides a summary and conclusions from the conducted literature review. This chapter summarizes the acquired knowledge regarding lightweight aggregates and the concrete produced from them. It highlights both the analyzed aspects and the areas requiring further consideration. Section 7, based on the information presented in the paper, verifies the validity of future research and its expected results. It also points out material analysis branches that need to be considered for a complete assessment of the validity of future research activities.

## 3. Lightweight Aggregate in Bridge Structures—Scientometric Analysis

Before delving into a detailed analysis of available works related to the design of bridges using lightweight aggregate concrete, a scientometric analysis was conducted based on publicly available library catalogs. This analysis aims to highlight the main trends in scientific research and the areas that require deeper analysis. Additionally, using the collected bibliometric data set, the most active authors and countries promoting the analyzed topic can be identified. Information regarding publication sources, the citation of scientific works, and the size of other bibliometric indicators allows for an initial verification of the significance of the described research. Such analyses are commonly used by authors in various scientific fields, including construction materials [48,49] and bridge engineering.

Currently, authors have access to a significant collection of library catalogs that are promoted by various publishers. Below are the citation analysis results for the Scopus, LENS, and WorldCat databases. The Scopus database, promoted by Elsevier, features a collection of over 1.8 billion publications from various scientific disciplines [50]. This database also provides a rich set of catalog metrics to facilitate the search and promotion of publications. The LENS database, in addition to a rich collection of publications, also includes information about patent applications. This database includes a module for visualizing selected bibliometric data parameters. The third library catalog used is WorldCat, which collaborates with 71,000 libraries from 112 countries. In addition to articles, this database includes materials such as audio, video, and graphics.

The use of three extensive databases increases the detail of the analysis. The search was limited to scientific articles, popular science publications, and books.

This approach ensures that the analysis is based on verified sources. Scientific journals and peer-reviewed publications eliminate unreliable and erroneous results. The multi-stage verification process guarantees high data quality. The results of this analysis are presented in Table 1.

**Figure 2:**
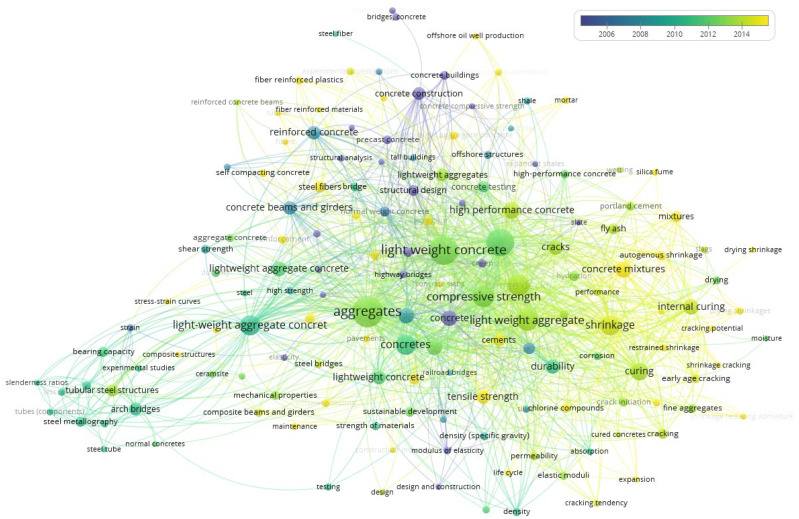
Visualization of the incidence and relationship of individual keywords (accessed on 30 January 2025).

Using the set of keywords “concrete bridge aggregate lightweight” a collection of publications was obtained for each library catalog. Subsequently, with the aid of VOSviewer software (version.1.6.20), a visualization was generated that illustrated the frequency of occurrence and relationships among the respective keywords for publications from the Scopus database (Figure 2). To present these findings in a readable manner, a minimum number of connections between keywords was set to 5, thereby reducing the total number of keywords from 1891 to 152. Based on the obtained visualization, strong connections were observed with the fields of materials science, concrete technology, and bridge engineering. Within materials science, topics related to production and physicochemical parameters of lightweight aggregates were prevalent. The appearance of the keywords “Ceramsite” and “expanded” highlights the significant use of expanded ceramics in lightweight aggregate bridge construction.

In concrete technology, research focuses on the preparation, curing, and final strength of concrete mixtures. This indicates the complexity of designing, producing, and maintaining lightweight aggregate concrete. Notably, steel fibers and fine powders are often used as additives. Additionally, self-compacting concrete with lightweight aggregate has been explored. These findings emphasize the necessity of precise design, execution, and curing processes for lightweight aggregate concrete.

In bridge engineering, studies primarily address the design and functionality of bridge structures. However, due to the complexity of construction and the absence of natural obstacles requiring weight reduction, standardized design guidelines remain limited. Furthermore, there is a notable absence of references to specific types of lightweight aggregates or the use of recycled materials. This is because aggregates used in bridge construction must be of natural origin, preventing the inclusion of waste-based binders in lightweight aggregates. This gap highlights a potential research niche for further investigation.

A color-coded analysis of keyword occurrences over time further illustrates research trends. The applied color scale assigns blue to the earliest keywords and yellow to the most recent. This pattern reflects ongoing research, particularly in concrete technology and the physicochemical properties of aggregates. Further advancements in this field will help validate the feasibility of lightweight aggregate concrete for bridge construction.

**Figure 3:**
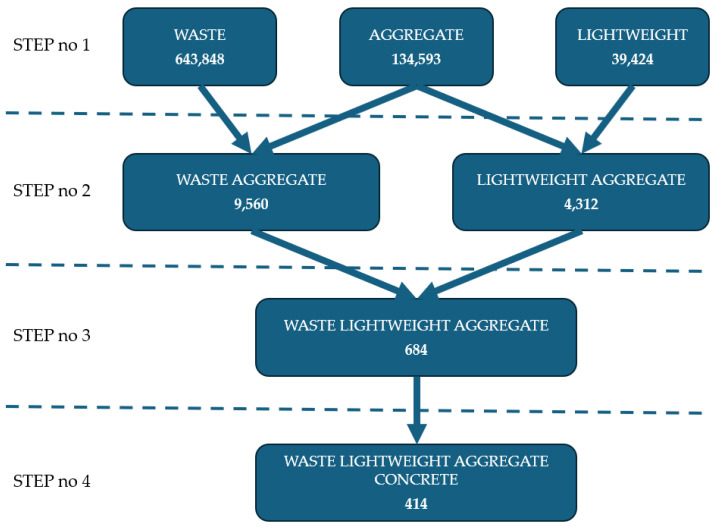
Graphical representation of the number of occurrences of the keywords “Waste lightweight aggregate concrete” based on data from the Scopus database (accessed on 30 January 2025).

Due to the lack of keywords directly related to the use of concrete with added waste in bridge engineering, a simplified analysis was conducted using the Scopus database. The aim of this analysis was to determine the number of studies focused on the application of lightweight waste aggregates in concrete production. The analysis was based on identifying the number of articles containing specific combinations of selected keywords. The various keyword combinations and the corresponding number of publications are presented in Figure 3. To ensure a broader scope of the search, keywords were analyzed not only within the keyword section but also in abstracts and titles. The results of the Scopus database analysis indicate that the topic of lightweight waste aggregates and their application in concrete remains a niche research area. A noticeable decline in the number of publications was observed as the number of keywords increased. Therefore, further research is needed to develop waste-based aggregates with high technical performance, making them suitable for bridge construction.

**Figure 4:**
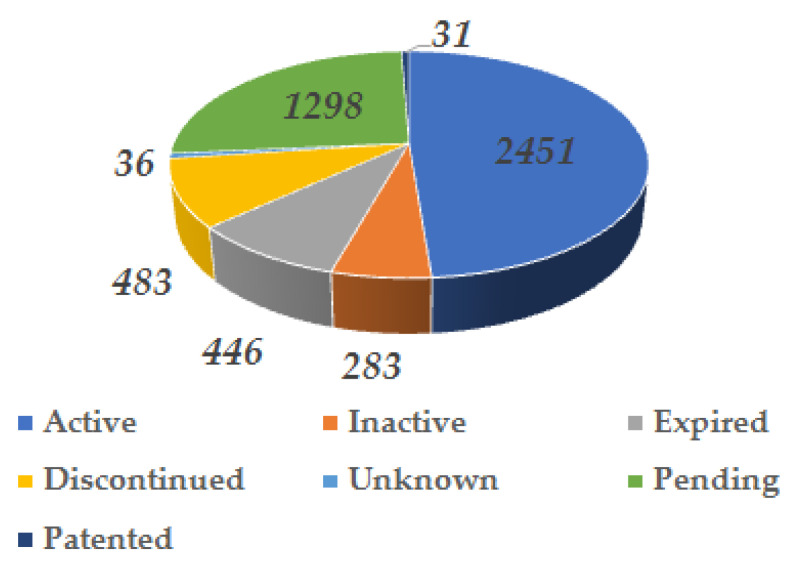
Summary of patents by legal status (accessed on 30 January 2025).

Based on the provided keyword set “concrete bridge aggregate lightweight” the content of the international patent database obtained from the LENS library catalog was examined. The data analyzed was acquired from the LENS catalog in January 2025. The database analyzed consists of 5028 patents, with over 49% of them still under active legal protection. Additionally, approximately 26% of the entire database analyzed comprises patents awaiting review. A detailed breakdown of patents based on their legal status is presented in Figure 4.

**Figure 5:**
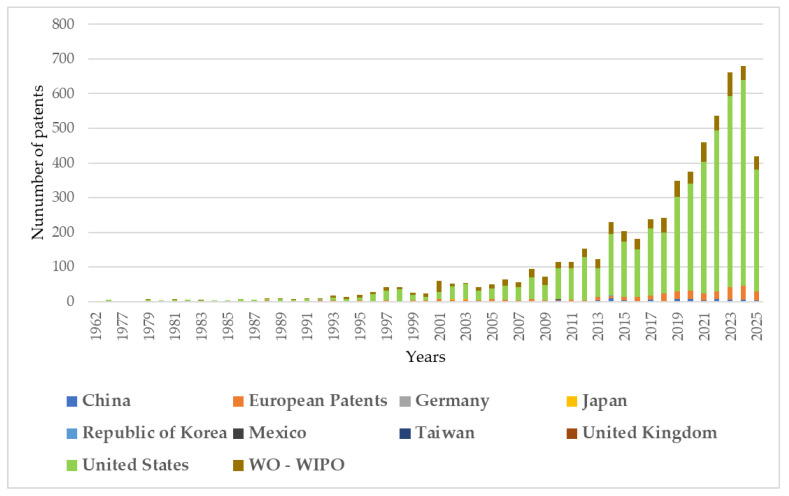
Graphic representation of the number of patents granted per year by country (accessed on 30 January 2025).

According to the LENS database analyzed, the vast majority of patents, specifically 3917 of them, have been published and registered in the USA. There are also notable numbers of patents from the World Intellectual Property Organization (WIPO), European Patents, and China, with 721, 312, and 66 patents, respectively. A detailed breakdown of the number of patents per country in relation to the grant dates is presented in the graph in Figure 5. The increasing trend in the graph confirms the growing interest in the subject analyzed over the last 10 years.

Analyzing the results from three independent bibliometric databases reveals that the use of lightweight aggregates in bridge engineering is an area requiring further research. Currently available studies on lightweight aggregates in construction primarily focus on basic mechanical parameters, such as the compressive strength and weight reduction of concrete. Topics related to the rheology of concrete mixes and hardened concrete are rare and have appeared only sporadically in the last decade. There is also a lack of research into the effects of destructive environmental factors on the performance of lightweight aggregates used in structural concretes. This limitation is due to the challenges of obtaining research specimens from bridge structures that are over 50 years old. Such studies could confirm the validity of previous research based on design standards. It is also noteworthy that there has been an increase in the number of patents filed in the area of “lightweight concrete bridge aggregates”. This trend suggests that patented solutions may be developed more frequently for industrial implementation in the future.

A simplified analysis based on the keyword set “Waste lightweight aggregate concrete” outlines the overall development in this area. It highlights the current direction of research aimed at developing eco-friendly materials made from waste. These studies primarily focus on creating lightweight aggregates from waste materials that are resistant to environmental factors. However, there is a lack of publications discussing the use of lightweight waste aggregates in producing structural concrete. Given that bridge structures are particularly vulnerable to environmental loads and require high durability, there are currently no examples of using lightweight waste aggregates for concrete production in bridges. A primary motivation for this situation is likely environmental protection, achieved through the reduction in natural aggregate extraction, the reuse of waste materials, and the reduction in the self-weight of bridge structures.

## 4. Detailed Analysis of the Current State of Knowledge Regarding Concrete Structures That Use Lightweight Aggregates

The analysis should begin with the definition of lightweight aggregates. Depending on their origin, lightweight aggregates can be divided into natural and artificial types.

Natural lightweight aggregates such as pumice, diatomite or sawdust are extracted from natural deposits. They are extracted and processed in the same way as normal lightweight aggregates. As a result, these types of aggregates typically have an angular and sharp surface structure. The arrangement of both external and internal pores is random, making it difficult to estimate their physico-mechanical parameters.

On the other hand, artificial lightweight aggregates are obtained by mechanical processes. They are most produced by sintering. In this process, pores can be created by burning combustible additives (such as polystyrene or sawdust) or by trapping exhaust gases. The key factors in the production of artificial lightweight aggregates are both the composition and the firing process to produce aggregates with the desired properties. Artificial lightweight aggregates are used in bridge construction due to their stabilized parameters. One of the significant drawbacks of lightweight aggregates is their lower compressive strength, especially when compared to natural aggregates. The presence of pores within lightweight aggregates, resulting from their production process, contributes to this lower strength. One approach to enhance strength, especially in uniaxial compression tests, involves incorporating steel [51] or polypropylene fibers [52,53].

This chapter presents the available lightweight aggregates and compares their physicochemical parameters with those of the natural aggregates that are currently used. The second part of the chapter includes existing guidelines concerning the design of lightweight concrete mixtures for use in bridge constructions.

### 4.1. Types of Lightweight Aggregates and Their Physical and Chemical Parameters

Pumice is a natural aggregate that has been used since ancient times. It played a role in constructing structures such as the Pantheon’s dome [25,54] and the Colosseum. The aggregate that forms during volcanic eruptions is considered amorphous aluminum silicate glass. As the pyroclastic material is released to the surface, a rapid process of glass formation occurs, along with gas release. This results in a material with a foamy structure [55]. Due to its natural origin, the detailed description of pumice’s composition and structure is unique and dependent on the source location. Before use, pumice needs to undergo mechanical processing, including crushing and grading into suitable particle sizes. The grading is carried out according to the guidelines outlined in relevant design standards [20,56,57,58,59]. Despite this, and due to the rapid foaming process, aggregates of this type have low density and low compressive and splitting strength. The fundamental parameters of lightweight aggregates obtained from processed natural pumice are provided by the manufacturer’s specifications [60,61].

Once prepared and graded, lightweight pumice aggregate can be used for producing lightweight concrete. Hossain’s work [62] studied the impact of replacing Portland cement CEM I with volcanic ash. Natural pumice from the Papua New Guinea region was used as an aggregate. The results indicated that with an increased percentage of volcanic ash, both compressive strength (initially 40 MPa to 22 MPa) and splitting strength (initially 3.7 MPa to 2.1 MPa) decrease. However, the addition of volcanic ash reduces the sample’s density by 25%. This addition also enhances the interfacial bond between the grain and the cement and also reduces drying shrinkage. A reduction in the self-weight of concrete based on natural pumice is also reported in [63]. The study confirms a decrease in the average compressive strength of concrete from 42.8 MPa to 19.1 MPa, and a decrease in tensile strength from 2.5 MPa to 1.9 MPa. Nonetheless, it is noted that the reduction in tensile strength, along with a reduced self-weight from 2395 kg/m^3^ to 1650 kg/m^3^, reduces the structure’s susceptibility to seismic loads. Muralitharan and Ramasamy [64] explored how the values of material parameters change as the percentage of natural pumice aggregate increases. The results confirm previous findings that as the percentage of lightweight aggregates increases, compressive strength decreases from 38.4 MPa to 6.86 MPa. Consequently, the best reduction ratio of compressive strength to mass was achieved with a 50% percentage of natural pumice. The research in [65] investigates the effect of changing the proportion of fine pumice aggregate and cement on the properties of compressive strength and thermal insulation. As the proportion of fine pumice aggregate in the mix increases, the thermal conductivity of the concrete decreases, as does the compressive strength under uniaxial compression (from 26.09 MPa to 14.63 MPa). One proposal for using volcanic-origin lightweight aggregate in cellular concrete is presented in [66]. The author studied concrete mixtures with varying percentages of lightweight pumice aggregate, and also examined their compressive strength, splitting tensile strength, and flexural strength. The results confirmed that as the percentage of lightweight aggregate in the concrete mix increases, compressive, splitting, and flexural strengths decrease. However, importantly, concrete with pumice aggregate demonstrated reduced abrasion depth in the abrasion test (using the Bohme disk) with an increasing percentage of lightweight aggregate. The potential to enhance lightweight pumice aggregate concrete using fibers is presented by Badogiannis et al. in [67]. The study created a concrete mixture based on Portland cement CEM IV/B and natural pumice aggregate, and this base mixture was blended with varying amounts of steel or polypropylene fibers. The results indicate that using pumice aggregate increases compressive strength by around 76% and flexural strength by about 110%. The impact on the modulus of elasticity and Poisson’s ratio was found to be negligible. Meanwhile, Hossain [68] analyzed the resistance of concrete mixtures that incorporate volcanic ash and pumice to sulfate and chloride exposure. The results show that mixtures based on volcanic ash and pumice exhibit less reduction in tensile strength when exposed to sulfate and chloride when compared to mixtures with Portland cement CEM I.

Table 2 provides a summary of the parameters of the mixtures described above based on natural pumice aggregate. Based on this, the use of pumice can be justified to reduce the overall weight of concrete. However, this aggregate can only serve as a partial substitute. A complete replacement of aggregate with pumice drastically affects the strength parameters of the concrete. Additionally, the variable density values of pumice indicate a lack of stability in the parameters of the lightweight aggregate.

**Table 2:** Summary of the obtained mixtures based on pumice aggregate.

Authors of the Papers	Name of Lightweight Aggregate	Bulk Aggregate Density [kg/m^3^]	Density [kg/m^3^]	Type of Cement	Compressive Strength [MPa]
Hess Pumice [60,61]	coarse pumice aggregate (Idaho, USA)	689	1426	N/A	16.9
	fine pumice aggregate (Idaho, USA)	785			
Hossain [62]	coarse pumice aggregate (Papua Nowa Gwinea)	680	1802–2520	Portland CEM I + volcanic ash	21–35
Muralitharan [63]	coarse pumice aggregate (Turkey)	870	1644–1656	Portland CEM I 52.5 + volcanic ash	18.66–19.11
Karthika [64]	coarse pumice aggregate (Turkey)	480	N/A	Portland CEM I 42.5	12.2–6.86
Hossain [68]	coarse pumice aggregate (Papua Nowa Gwinea)	1870	N/A	Portland CEM I, V + volcanic ash	25–38
Badogiannis [67]	coarse pumice aggregate	N/A	1710–1840	Portland CEM IV/B (P-W) 32.5R	18.56
Gündüz et al. [65]	coarse pumice aggregate (Turkey)	815–935	1150–1271	Portland CEM I 42.5	14.63–26.09
	fine pumice aggregate (Yali Island)	895–925			
Öz [66]	coarse pumice aggregate	990	1206–1886	Portland CEMI 42.5R	13.69–7.04

A separate category of lightweight aggregates consists of aggregates formed from mineral raw materials subjected to thermal and mechanical processing. Aggregates of this type are classified as artificial lightweight aggregates.

One of the first aggregates of this kind was Haydite aggregate [29]. Its production involves using clays or shales, along with foaming agents. The raw material is subjected to granulation and then fired in a kiln at a temperature of about 1100 °C. During the firing process, the raw material expands due to carbon oxidation and the release of gas [69]. The initial research on the use of the lightweight aggregate Haydite in concrete production covered the impact of the aggregate on the concrete’s parameters [70]. Based on these studies, the optimal percentage of lightweight aggregate in the mix was determined. Richart et al. demonstrated that with a 55% proportion of lightweight Haydite aggregate in the concrete mix, the ratio of its own weight to compressive strength would be the smallest.

The first studies describing the scope of applying lightweight aggregates and estimated strength parameters date back to the 1950s. Based on data presented in [69] the fundamental advantages of Haydite lightweight aggregate can be indicated, including a 30% reduction in self-weight compared to normal aggregates. Furthermore, an increased resistance of lightweight aggregate concrete to fire was noted. The mentioned study also provides the proposed concrete mix compositions, the parameters of which are presented in Table 3. Studies conducted by the Iowa State Highway Commission [71] aimed to develop concrete mix compositions using artificial lightweight aggregates. These mixes were intended as the basis for the concrete used in road construction. Within the framework described in [71], concrete mixes were prepared using three types of lightweight aggregates: Haydite, Idealite, and Materialite. The requirements regarding strength parameters and frost resistance of the lightweight concretes used in road construction were met by the mixes containing Haydite and Idealite aggregates. The parameters of the individual concrete mixes with lightweight aggregate are presented in Table 3.

Wang et al. [72] conducted detailed tests on samples made from lightweight concrete subjected to axial compression by analyzing the three-dimensional stress state. Haydite lightweight aggregate was used as the lightweight aggregate. The obtained test results indicate a different failure model when compared to samples made from natural aggregate concrete. As a result, a novel four-parameter criterion for estimating the three-dimensional failure state of lightweight concrete under axial compression was proposed. In turn, work [52] describes research on the influence of adding rubber and polypropylene GRT fibers on the strength parameters of lightweight concrete using Haydite lightweight aggregate. Based on the obtained results of elastic modulus and concrete strength, a proprietary computational model was proposed. With the development of knowledge in the production and physicochemical parameters of lightweight aggregates, attempts have been made to influence the composition of the raw material mixture used in lightweight aggregate production. Zhang et al. [73] presented the concept of using sewage sludge for the production of lightweight Haydite aggregate. This aggregate is characterized by smaller internal pores. The different pore structure in the modified lightweight aggregate results in a lower water absorption by 0.6% and greater cylindrical compressive strength by 6.6 MPa when compared to the unmodified lightweight aggregate. Chen and Zhang [74] conducted tests with varying percentages of sewage sludge in the aggregate composition. The described study focused on the influence of heating temperature and sintering on the basic strength parameters of lightweight aggregates containing waste. The obtained results indicate that the best results for water absorption and crushing strength of modified lightweight aggregates were obtained by initially heating the aggregate and sintering it at temperatures up to 600 °C and 1100 °C, respectively. For this reason, it is recommended to extend the firing process of modified lightweight aggregates with the addition of sewage sludge.

Due to its properties, Haydite lightweight aggregate has also found applications in the construction of bridges in the United States, as described in Chapter 5 [26,75]. This demonstrates the validity of using lightweight aggregates in the production of structural concrete for bridge construction. With a detailed analysis of the aggregate composition, it is possible to design bridges using it.

Analyzing a number of publications, it is important to note the fundamental advantages of artificial lightweight aggregates. These advantages include the stabilization of strength parameters and their own density. As a result, it is possible to obtain concrete with a predictable final strength. Additionally, studies describing the use of sewage sludge for the production of lightweight aggregates represent an interesting area of development. Sewage sludge is used as a foaming agent for the aggregate during firing. This indicates the potential for future research in this area. A key aspect remains the issue of compounds formed during the firing process of the aggregate. Therefore, the production of artificial lightweight aggregates with waste additives as a foaming agent could represent a promising direction for research.

**Table 3:** The compilation of mixes using Haydite aggregate.

Authors of the Papers	Name of Lightweight Aggregate	Bulk Aggregate Density [kg/m^3^]	Density [kg/m^3^]	Type of Cement	Compressive Strength [MPa]
Richart et al. [70]	fine Haydite aggregate	865	1490–1698	Portland type I	12.2–31.1
	coarse Haydite aggregate	689			
Iowa State Highway Commission [71]	coarse Haydite aggregate	855–924	N/A	Portland type I	46.3–47.1
Muralitharan [63]	coarse Idealite aggregate	887	1644–1656	Portland CEM I 52.5 + volcanic ash	50.0–54.9
Karthika [64]	coarse Materialite aggregate	847	N/A	Portland CEM I 42.5	44.5–46.1
Wang et al. [72]	coarse Haydite aggregate	693	1531	Portland type I	47.38–58.86
Xia et al. [52]	coarse Haydite aggregate	800	1856–1942	Portland type I 42.5	21.82–34.91

One of the most popular lightweight aggregates in the European region is Liapor LECA Ceramsite. The popularity of LECA Ceramsite lightweight aggregate is due to the widespread availability of the raw materials it is made from, namely clay and shale. Additionally, the production process is well-defined, ensuring the consistency of the aggregate’s parameters. Roces et al. [76] provided a detailed description of the structure, density, and water absorption of LECA lightweight aggregate. It is worth noting that their proposed water absorption model assumes that the aggregate will achieve 90% saturation in 15 months, and complete saturation over an infinite immersion time.

Ceramsite is used for reinforcing and stabilizing slopes and road embankments, as well as the foundations and soils beneath foundations. It can also serve as drainage fill (Figure 1). In Poland, only a few plants are engaged in the industrial-scale production of ceramsite. One of them is located in Mszczonów in the Mazowieckie Province. The fundamental physico-chemical parameters of this aggregate are presented in [77]. The use of glauconite additives improves the texture of the sinter and increases the porosity of the aggregate itself.

Research on lightweight concrete using ceramsite aggregate was described by Wegian in [78]. The author developed concrete mixes with varying proportions of ceramsite, sand, gravel, and dolomite aggregate to compare their compressive strength, tensile strength, workability, and density. The density of ceramsite concrete ranged from 71% to 75% when compared to the density of mixes with natural aggregate. Ultimately, the highest compressive strength values were achieved for concrete samples containing fine lightweight aggregate. In turn, Issa et al. [79] presented the results of comprehensive research on concrete based on lightweight Ceramsite aggregate. The obtained compressive and tensile strength values, as well as the density, classify the concrete as structural. Details regarding the parameters of the proposed concrete mixes are presented in Table 4. The concept of reinforcing various types of lightweight concretes with fibers also applies to expanded shale aggregates [53,80]. The presented studies confirm the effectiveness of using hybrid steel and polypropylene fibers to increase flexural and splitting strength by 57% and 35.5%, respectively. Furthermore, concrete with fiber demonstrates higher ductility, which in turn affects energy absorption capacity. In the second mentioned study, Wu et al. [80] confirmed the increase in flexural and splitting strength by adding steel and carbon fibers. The greatest increase was achieved with a volume ratio of steel and carbon fibers equal to 0.9% of the concrete sample’s mass. The fibers themselves also affect the compressive strength of concrete, although to a small extent. Zhao et al. [81] conducted an analysis of the impact of steel fibers on the shrinkage of lightweight concretes. The study utilized both lightweight ceramsite aggregate and lightweight sand. The conducted shrinkage measurement revealed faster and greater growth with regard to aggregates with lower compressive strength. Additionally, the effectiveness of using fibers to reduce autogenous and drying shrinkage was confirmed. The impact of moistening lightweight expanded clay aggregate on concrete mix parameters was presented by Lo et al. in [82]. It is noteworthy that the maximum compressive strength for lightweight aggregate concrete is achieved with lightweight aggregate that has been pre-moistened for 30 min. The research in [83] presents a compilation of the basic physico-chemical parameters of lightweight concretes using ceramsite and expanded shale aggregate with the addition of fly ash. The research included measurements of shrinkage, compressive strength, elastic modulus, and freeze–thaw resistance.

When analyzing lightweight aggregate concretes (LWC), special attention should be paid to the mix’s behavior under the influence of chloride compounds. These substances, commonly found in de-icing agents, pose a significant threat to concrete bridge structures. According to the findings presented in [84], the chemical resistance of lightweight aggregates to chloride aggression is comparable to, or in some cases even exceeds, that of natural aggregates.

Hornáková et al. [85] conducted a comprehensive study on lightweight concretes reinforced with steel fibers. The research included variations in fiber content to assess the influence on both mechanical performance and durability. In addition to standard mechanical tests, surface electrical resistivity measurements were performed [86], providing insights into the concrete’s permeability. The results indicated an increase in the diffusion coefficient of chloride ions with higher fiber content. This observed relationship enabled a numerical analysis of chloride penetration at varying concentrations and its impact on the mechanical properties of LWC [87]. Furthermore, approximate analytical expressions were developed to predict the deterioration trends of fiber-reinforced LWC exposed to chlorides [88].

Fire tests on mixes based on lightweight aggregate, described in [89], demonstrate their effectiveness when compared to regular concrete. The residual compressive strength of lightweight concretes after exposure to thermal loads was greater when compared to regular concrete within the temperature range below 300 °C. Within the range of 300–800 °C, the behavior of both concretes was similar. Yao et al. [90] compared the residual strength of lightweight concretes with regard to the proportion of lightweight ceramsite aggregate. The presented results indicate significantly improved fire resistance in comparison to traditional mixes. It is worth noting that at a thermal load of 1000 °C, concrete based on lightweight ceramsite aggregate exhibits a reduction in compressive strength by up to 80%. Regular concrete based on natural aggregates loses over 95% of its compressive strength at a thermal load of 1000 °C.

An interesting proposal appears to be cylindrical ceramsite aggregate, which was presented in [91]. The innovative aggregate production method ensures an extremely lightweight aggregate with a porous structure. The aggregate itself has a crushing strength of 0.87 MPa and a density of 375 kg/m^3^. The authors’ acknowledgment of the need for further research in the context of the obtained low crushing strength values indicates the necessity of refining the aggregate’s composition and production process. Within the aforementioned plant, research was also conducted on the possibility of using sewage sludge for lightweight aggregate production [92]. The application of sewage sludge is intended as a thickening additive required for the production of expanded aggregates. The resulting aggregate is characterized by good insulation properties and significant absorbency. The intended use of the designed aggregate is in the production of lightweight thermal insulating concrete.

The Nanjing Yangtze River Bridge in China, built in 1968, can also be seen as an impressive structure. The bridge deck was made using expanded clay aggregate. After 50 years of service, the bridge underwent modernization. Samples collected after the modernization allowed Huang and other researchers (Huang et al. [93]) to analyze the hydration products of the 50-year-old lightweight concrete, and to characterize the interfacial transition zone (ITZ). The studies confirmed the weakening of the concrete structure after 50 years of service and the susceptibility of the structure to degradation associated with cyclic temperature changes. Additionally, X-ray diffraction (XRD) and scanning electron microscopy (SEM) studies confirmed the ability of cement to penetrate the surface pores of the aggregate, leading to better bonding of the elements. The prepared research material also allowed for the production of concrete mixes using recycled lightweight aggregate and mortar [94]. The results obtained indicated the justification for the use of recycled lightweight aggregate.

The summary of the research results obtained is presented in Table 4. The number of studies confirms the popularity of this type of lightweight aggregate. The benefits arise from the stability of both the aggregate parameters and the mixture made from it. Similarly to Hayite aggregates, research is being conducted on the use of sewage sludge as a foaming additive. Notably, due to its popularity, this aggregate has been used in the construction of bridge deck slabs. This allows us to confirm the performance of concrete with lightweight aggregates under chloride aggression conditions.

**Table 4:** Summary of the mixtures using LECA Ceramsite aggregate.

Authors of the Papers	Name of Lightweight Aggregate	Bulk Aggregate Density [kg/m^3^]	Density [kg/m^3^]	Type of Cement	Compressive Strength [MPa]
Wegian [78].	fine/coarse Ceramsite aggregate	510–1480	1360–2390	Portland	11.8–32.2
Issa et al. [79]	coarse Ceramsite aggregate	415	1760–1824	Portland	18.0–30.5
Nemes et al. [89]	coarse Ceramsite aggregate	1047–1380	N/A	Portland CEM I 42.5 N/52.5 N	43.5–78.0
Yao et al. [90]	coarse Ceramsite aggregate	415	1679–2167	Portland CEM I 42.5	14.2–30.7
Wei et al. [53]	coarse Ceramsite aggregate	860	1724–1849	Portland cement P. O 42.5	41.0–67.0
Wu et al. [80]	coarse Ceramsite aggregate	755–1019	1720–1940	Portland cement P. O 42.5	47.0–86.0
Zhao et al. [81]	coarse Ceramsite aggregate	816–888	1695–1946	Portland cement P.O. 52.5	47.2–64.8
Pichór et al. [92]	coarse Ceramsite aggregate	725–785	1500–1534	Portland Cement CEM II 32.5	16.3–18.1
Lo et al. [82]	coarse expanded clay aggregate	405	1617–1851	Portland Cement	29.19–42.95
Mohammed et al. [83]	coarse shale aggregate	1784	N/A	Portland type I	25.7–44.8
	coarse clay aggregate (Arcosa)	1590	N/A		32.1–47.7

Artificial lightweight aggregates can also be made from fly ash, which is a byproduct of the combustion process. Utilized dusts containing trace amounts of unburned fuels, along with the addition of coal sludge, can form the basis for further granulation processes. An example of this type of aggregate is Lytag aggregate, derived from the fly ash from power plants. The concept of producing such aggregates emerged in the late 1950s in the United Kingdom [95]. Currently, this type of aggregate is produced throughout Europe. The physico-chemical parameters of ash-based aggregates are presented in the manufacturer’s guidelines [96,97,98]. Guidelines for designing lightweight concrete using Lytag aggregate are presented in works [99,100,101], which summarize the design guidelines from standards [17,19,102].

The pioneering research presented in [103] describes the structure of aggregates based on fly ash. This aggregate is characterized by a darker interior that is surrounded by a light outer layer. It is important to emphasize that both layers are closely connected. The pores within the aggregate have a honeycomb-like structure. Due to this construction, the aggregate exhibits high freeze–thaw resistance and high water permeability properties. The presented research defines the absorption of the aggregate at a level of 13%. The work presented by Zhang [104] includes a description of the basic mechanical parameters of concrete using lightweight Lytag aggregate. Based on the obtained research results, the author developed relationships between, for example, the compressive strength of cubic samples and cylindrical concrete samples. Ahmad et al. [105] describe the influence of increasing the percentage of lightweight aggregate on the strength parameters of concrete mixes. The highest strength value under uniaxial compression, while maintaining reduced self-weight, was achieved by concrete samples with a 50% content of lightweight Lytag aggregate. Comprehensive research on the impact of combustion-derived materials on concrete mixes is presented in [106]. The study examines the influence of fly ash and Lytag aggregate on the parameters of both the concrete mix and solidified concrete samples. As a result, concrete mixes with a self-weight below 1990 kg/m^3^, and compressive strength ranging from 20 to 40 MPa, were obtained. Similar results regarding the reduction in compressive strength were obtained in the studies by Punlert et al. [107]. The aggregate used in the study contained various proportions of clay and fly ash, and was fired at different temperatures. Ultimately, the smallest ratio of the self-weight to compressive strength of the aggregate was achieved for a mixture of clay and ash in the ratio of 80:20%. The firing process of the aggregate was carried out at 1210 °C. The parameters of the resulting lightweight concrete are presented in Table 5.

In turn, Domagała [108] conducted research on the influence of the formulation of lightweight concrete mixes on water absorption, water permeability, and resistance to freeze–thaw cycles. The research considered the influence of the initial moisture content of the aggregate, particle size distribution, and water-to-cement ratio w/c. As a result, concretes with densities ranging from 1470 to 1920 kg/m^3^ and compressive strengths ranging from 25.0 to 83.5 MPa were obtained. Importantly, the need to limit the initial moisture content of the aggregate and to choose a tight cement matrix was noted. These actions directly affect the durability of the final mix. Research confirming freeze–thaw resistance is presented in [109]. Zhang et al. [109] confirmed the suitability of using lightweight aggregate with cementitious combustion products like fly ash or slag. Due to its porous structure, lightweight aggregate is resistant to freezing/thawing and salt exposure.

An example described in [51] involves research aimed at improving the strength of concrete based on lightweight aggregates from sintered fly ash with the use of steel fibers. In order to determine the scale of the fiber’s influence, two mixes were prepared with three fiber doses. As a result, using steel fibers in an amount of 0.8% of the volume of concrete increased the concrete’s splitting and flexural strength by 73% and 61%, respectively.

It is worth noting that a series of studies confirms the acceptance of lightweight concrete as a structural material for bridge structures. For example, guidelines [98] estimate the potential self-weight reduction of a span above 100 m by 24%. The use of lightweight aggregates in bridge structures has been allowed in the design standard [102]. Confirmation of this state of affairs can be seen in research on bridge elements using lightweight aggregate [110,111], and also in existing structures [112].

In the summary, it is important to highlight that Lytag aggregate is derived directly from waste processing. This underscores the validity of using waste materials for the production of lightweight aggregates. The resulting aggregate exhibits stable mechanical properties. Numerous studies analyzing the behavior of this aggregate at low temperatures should also be noted, as they illustrate the aggregate’s performance under natural conditions.

**Table 5:** A compilation of concrete mixes with Lytag aggregate.

Authors of the Papers	Name of Lightweight Aggregate	Bulk Aggregate Density [kg/m^3^]	Density [kg/m^3^]	Type of Cement	Compressive Strength [MPa]
Lytag [99,100,101]	coarse Lytag aggregate	750–850	1400–1800	Portland CEM I	22–50
Zhang et al. [109]	Lytag 800 (Chiny)	N/A	N/A	Portland typ I 42.5	37.8–42.2
Ahmad et al. [105]	coarse Lytag aggregate	687	1879–1954	Portland typ I	36.6–37.7
Yun Bai et al. [106]	coarse Lytag aggregate	1520	1559–1977	Portland typ I 42.5N	20–40
Punlert et al. [107]	coarse Lytag aggregate	1660	1780–2210	N/A	40.94–42.34
Domagała [108]	coarse Lytag aggregate	720–730	1470–1920	Portland cement CEM I 42.5 R	25–83.5
Zhang [104]	coarse Lytag aggregate	794	1490–1940	Portland typ I 42.5	28.9–50.2
Domagała [51]	coarse Lytag aggregate	N/A	1580–1710	Portland cement CEM I 42.5 R	39.0–47.5

Another equally interesting proposition is the utilization of processed waste as lightweight aggregates. Pordesari et al. [113] conducted research on the utilization of coconut shells as lightweight aggregate in concrete. The obtained mixtures exhibited better workability and a reduction in density of up to 15% when compared to concrete based on granite aggregate. The low density of the aggregate is associated with a lower compressive strength and propagation of cracks, along with a decreased modulus of elasticity. As waste lightweight aggregate, it can be sourced from agricultural waste [114] as well as from construction [115] or industrial [116] waste. Due to the long history of using lightweight aggregates in construction, research is also being conducted on their reuse from demolished buildings. Wongkvanklom et al. [117] investigated concrete mixtures by replacing limestone aggregate with recycled lightweight aggregate (RLA). RLA comprised crushed post-production waste from autoclaved concrete. The research results indicate the possibility of replacing up to 35% of natural aggregate with RLA, while at the same time maintaining strength parameters compliant with ASTM C330/C330M standards [20], in turn enabling the fulfillment of the requirements for structural lightweight concrete. In contrast, Li et al. [118] and Kashyap et al. [119] utilized aggregate recovered from the demolition of structures built using lightweight aggregate. Concrete containing lightweight aggregate was used in the studies. The research results suggest that with the inclusion of 30% aggregate from waste, the decrease in compressive strength of concrete compared to the reference sample is minimal or non-existent.

One of the benefits often overlooked in lightweight aggregates is their capacity for water retention and the internal sealing of structures. The paper [120] describes a study that confirms the effect of lightweight aggregates on the sealing of concrete. Furthermore, detailed descriptions of bridge structures where lightweight aggregates were employed for internal sealing are provided in the paper. According to work [121], for the same strength, lightweight aggregate concrete (LAC) with a higher moisture content of lightweight aggregates (LWAs) exhibits reduced early-age shrinkage, but ultimately experiences greater shrinkage when compared to normal-weight concrete (NWC). However, LAC with a lower moisture content of LWAs consistently exhibits higher shrinkage than NWC. When a portion of LWAs with a lower water absorption is replaced with crushed limestone, the shrinkage of LAC could be reduced.

With the advancement of lightweight aggregates, the concept of developing concrete with the strength parameters of high-strength concrete and the benefits of lightweight concrete has emerged. This has led to the development of lightweight concrete mixes with high strength. Studies [122,123] summarize previous research on such concrete mixes in order to establish guidelines for designing high-strength lightweight concrete mixes. By using the accumulated knowledge, it is now possible to create concrete mixes with strengths exceeding 100 MPa and volumes ranging from 1995 to 2114 kg/m^3^ [124]. Additionally, in the context of high-strength lightweight aggregates, the influence of steel fibers on concrete strength and shrinkage was considered. Based on research presented in [125], a reduction in shrinkage can be observed with an increase in the proportion of steel fibers up to 1.5% of the mix. Importantly, high-strength lightweight concrete exhibits a higher final shrinkage when compared to mixes using conventional aggregates.

### 4.2. Principles of Structural Design for Structural Lightweight Concrete

Designing the composition of the concrete mixes used in the construction of bridge structures requires the consideration of various factors. Among these, European standards highlight compressive strength, exposure class, consistency class, maximum aggregate size, and chloride content as key parameters [126]. For lightweight aggregate concrete, information about the mix density is also essential.

The following subsection provides an overview of guidelines for designing structures using lightweight aggregate concrete, with a specific focus on recommendations for bridge structures.

#### 4.2.1. United States Design Guidelines

Due to the extensive history of designing and constructing bridge structures using lightweight aggregate concrete, the United States has the most comprehensive guidelines for designing lightweight concretes. A significant portion of these guidelines originate from the states of Virginia and Alaska.

One of the initial guidelines regarding lightweight aggregate concrete was included in the ACI Committee 213 report of 1967 [59]. The guidelines define structural lightweight aggregate concrete as “Structural lightweight-aggregate concrete made with structural lightweight aggregate as defined in ASTM C 330” [20]. The compressive strength of the designed mix should be greater than 17 MPa, and the density should range from 1120 kg/m^3^ to 1920 kg/m^3^.

In Chapter 4 of the report [59], the compressive strength requirement is tightened to 35 MPa for prestressed structures and above 60 MPa for bridge structures, with bridges constructed in Norway and Japan being used as examples. The chapter also provides guidelines for estimating the modulus of elasticity, split tensile strength, shrinkage, and creep. Notably, Section 4.14 of the report [59], titled “Durability”, highlights freeze/thaw resistance. Based on the studies described in paper [127], the outstanding resistance of concretes (based on lightweight aggregate) to microcracking was confirmed. This resistance was facilitated by the arrangement of pores and the dense contact zone between the aggregate and mortar. In turn, the contact zone determines concrete permeability, mechanical strength, and chemical resistance.

Chapter 6 of the report [59] provides guidelines for designing high-strength lightweight concrete. Increased strength necessitates a density increase to above 1920 kg/m^3^; however, as a result, it is possible to achieve concrete mixtures with strength exceeding 60 MPa. Such concretes are used in projects like the construction or renovation of existing structures [127]. Detailed descriptions of these projects are included in Chapter 5.

The execution of prestressing in bridge structures using lightweight aggregate concrete is discussed in study [128]. Based on previously published works, the authors presented an analysis of prestressing in two reference concrete mixtures with compressive strengths of 41 MPa (6 ksi) and 55 MPa (8 ksi). The composition of the reference mixes is provided in Table 6.

Ultimately, mixtures with dry densities of 118 pcf (1890 kg/m^3^) and 122 pcf (1954 kg/m^3^) were obtained for the 6 ksi (41.37 MPa) and 8 ksi (55.16 MPa) reference mixes, respectively. Additionally, workability, modulus of elasticity, split tensile strength, creep, and shrinkage tests were conducted. The subsequent part of the study focused on the investigation of wave transmission lengths and strand dispersion in the developed concrete mixes and compared the obtained values with recommendations from [129,130].

Numerous examples of lightweight concrete usage in bridge engineering led to the creation of the AASHTO LRFD guidelines in 1998 [130], which have been updated to the present day [26,131]. According to these guidelines, concrete density must not exceed 2160 kg/m^3^ (0.135 kcf), and compressive strength should range from 35 MPa to 68 MPa (5–10 ksi). Many structures in the United States have been constructed based on these guidelines [132].

Other works related to bridge design mention lightweight aggregate concrete as an alternative for a prefabricating bridge segment [133].

Verification of the AASHTO LRFD assumptions [131] is presented in study [134]. Greene et al. compared material test results for lightweight aggregate concretes with values obtained based on the specified guidelines. Compressive strength, split tensile strength, and modulus of elasticity values were compared. The results obtained by them provide evidence of the conservativeness of the guidelines with regard to lightweight concrete design.

#### 4.2.2. European Design Guidelines

Within the European Union member states, unified design procedures have been introduced through standardized EN design norms, along with national annexes. The foundation for designing concrete mixes is the norm EN 206 “Concrete—Specification, performance, production, and conformity” [18]. This norm defines the concepts of lightweight concrete and lightweight aggregates. It also establishes the classification of lightweight concrete based on density, as presented in Table 7.

Regarding lightweight aggregates, the norm [18] references the standard PN-EN 13055 Lightweight aggregates [17]. This norm, along with accompanying standards [135], defines the suitability and parameters of lightweight aggregates.

In turn, PN-EN 1992-1-1 [19] and PN-S-10040 [136] provide guidelines for designing mixes intended for bridge structures. According to the recommendations of these norms, concrete should exhibit good workability, high compressive strength, and durability associated with concrete impermeability [137]. Detailed requirements for the concrete used in bridge construction are provided in Table 8.

The set of recommendations, currently considered outdated, permits the use of only volcanic natural aggregates in the construction of bridge structures. Consequently, lightweight bridge construction is not very common in Europe [139]. The prevailing assumption about the instability of lightweight aggregates due to their high absorption discourages attempts to execute such constructions.

In this regard, Norway stands out significantly, as it has been successfully using lightweight aggregates for years to lighten bridge structures and accelerate their construction and renovation processes [140]. The application of American design guidelines has enabled the creation of the structures described in Section 5.

It should also be emphasized that the current European guidelines allow for the use of structural lightweight concrete.

#### 4.2.3. China Design Guidelines

In the analysis of design guidelines applicable in China, it can be observed that the foundation for designing structures using lightweight aggregate concrete is based on guidelines from other countries [141], such as the United Kingdom and the USA [142]. Based on this, Chinese engineers developed the GB 2839-81 standard [143], which provides guidelines for designing and implementing structural lightweight aggregate concrete. Currently, the GB/T 20473-2017 standard [144] is used for designing structures using lightweight aggregates. Regarding the design of bridge structures, a division has been introduced for road bridges [145] and railway bridges [146]. Within this division, design standards were established that cover seismic loads [147] and turbulence caused by wind effects, among other factors. The standardization system in China is quite complex due to the multitude of institutions and associations involved in this area [148].

When comparing guidelines for the design of lightweight bridges, it is worth noting that the US guidelines are the most comprehensive in this area. They contain the necessary material information for the correct design and construction of a concrete mix based on lightweight aggregates. However, due to the specific nature of the climate, particular attention should be paid to the European guidelines in the context of environmental impact. In particular, the requirements for frost resistance and chloride resistance are important.

#### 4.2.4. Summary of Design Guidelines

The design guidelines presented above serve as the foundation for further research assumptions. Based on them, the minimum compressive strength of lightweight concrete and the threshold values of selected physicochemical parameters—such as bulk density, water absorption, frost resistance, and resistance to chloride compounds—have been defined. These guidelines will serve as a reference for future research focused on developing an original waste-based lightweight aggregate and concrete incorporating this aggregate.

## 5. Lightweight Aggregate Concrete in Bridge Structures

To support the validity of the scientific studies and normative guidelines, a review of previously constructed bridge structures that utilize lightweight aggregate concrete has been conducted. In the presented cases, lightweight concrete was used for purposes such as renovating and modernizing existing bridge structures, as well as for constructing new ones. It is worth noting that the showcased implementation examples represent a well-documented group of structures. This implies that the total number of lightweight aggregate concrete bridge structures worldwide is likely to be much more extensive.

The following section provides a description of examples that showcase the use of lightweight aggregate concrete in bridge engineering around the world. It also includes intriguing proposals concerning the utilization of lightweight concrete for future bridge constructions.

The use of lightweight aggregates in bridge structures mainly stems from the desire to reduce the overall weight of the structure. Lightweight concretes are often used to construct bridge decks [149,150,151], or the components between spans [152,153,154,155]. However, these structures are less frequently entirely made of lightweight aggregate concrete, which is since lightweight aggregates typically have lower mechanical parameters when compared to natural aggregates. Additionally, lightweight aggregates contribute favorably to increasing the resistance of the structure to dynamic loads [156,157].

Nevertheless, the use of lightweight concrete has certain disadvantages. Some issues, such as difficulties in pumping the mix [158,159], water absorption by the aggregate, or incomplete hydration of the concrete may arise. However, these drawbacks can be mitigated through proper preparation of the aggregate before the production of the concrete mix.

### 5.1. Bridge Structures in Japan

The concept of using lightweight aggregates for constructing concrete bridge structures emerged towards the end of the 1960s. One of the earliest publications discussing this approach comes from Japan [160]. Within these studies, various parameters were determined, such as the flow and workability of the mixture; compressive, tensile, and flexural strength; as well as shrinkage and creep of the concrete. Based on the obtained results, it was established that due to the porosity, absorptive properties, and weight of the lightweight aggregate, pumping the mixture could be challenging. Meanwhile, the weight was reduced by 20–30% and the Young’s modulus and shear strength values were decreased, but compressive and tensile strengths remained unchanged. Different combinations of the percentages of lightweight and natural aggregates were used in the research. The lightweight aggregate used was mesalite [161], which is calcined expanded shale. However, the work did not stop at laboratory tests alone. Based on the findings, two railway bridges were constructed over the Arakawa River [160]. These structures were erected on weak ground, necessitating a lightweight design resistant to ground settlement. Intermediate support of the bridges was made as lightweight reinforced concrete portal piers. Similarly, the Shiosai pedestrian and bicycle bridge, spanning 232 m, was predominantly constructed using prefabricated elements made with lightweight aggregate [161]. This four-span cable-stayed bridge, opened for use in 1995, follows the design of an “Inverted Suspended Bridge” [162,163]. In structures of this type, the load is transferred from the bridge deck to the supporting columns below. These columns distribute the load to a tensioned belt directly connected to the pylons or abutments.

Furthermore, Otsuka et al. [159] explored the use of high-strength lightweight concrete in the construction of the Touja Bridge in Kumamoto Prefecture. This work discusses the potential of pumping concrete mixtures to heights of up to 70 m vertically. Laboratory and on-site research conducted in this study confirmed the workability of the concrete mixture.

Another study [164] presents examples of using lightweight aggregates for producing high-strength concrete. In the introduction, the authors describe newly developed lightweight aggregates used in construction, including Perlite, Liapor, and coal ashes. The subsequent part of the study details research on mixtures made with Perlite-based lightweight aggregates [165], confirming the effectiveness of lightweight concrete mixtures. As a summary, the study presents selected examples of using lightweight aggregates in bridge structures both in Japan and abroad. For instance, the planned load reduction of the Sendai Hokubu Road bridge is highlighted. This three-span bridge, measuring 256 m in length, is situated on weak ground that is susceptible to earthquakes. The use of lightweight aggregates will lighten the structure and reduce construction costs.

**Figure 6:**
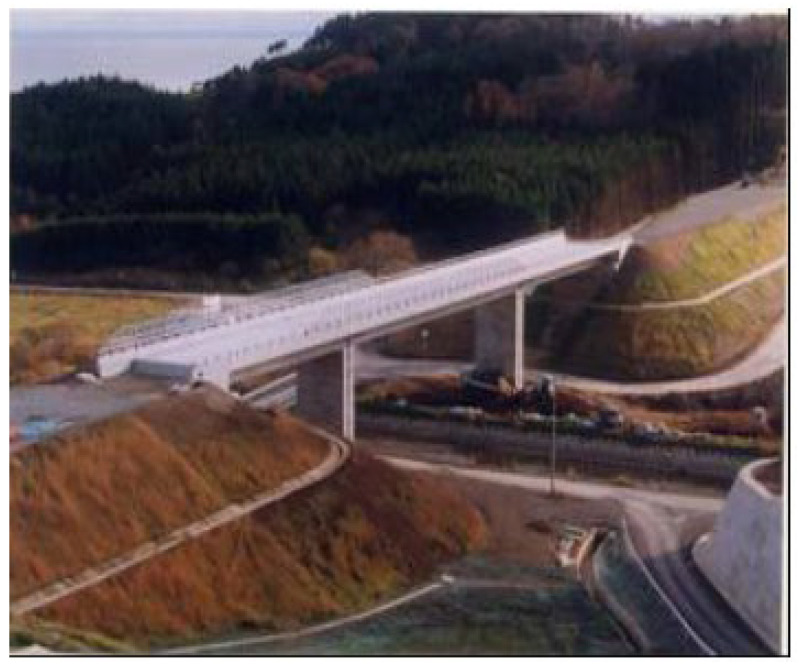
Shirarika bridge design [166].

Another example, the Shirarika Bridge in Japan (Figure 6), showcases the application of Perlite-based lightweight aggregates to achieve high-performance lightweight concrete [166]. This continuous three-span box-girder bridge is prestressed longitudinally with external tendons. The chosen lightweight aggregate exhibits higher strength and lower water absorption when compared to conventional lightweight aggregates [167].

The study presented in [168] introduces the concept of a railway bridge design using steel tubes filled with lightweight aggregate concrete. The research compared the effectiveness of various structural types in terms of flexural and shear strength, as well as noise reduction. In terms of flexural strength, the concrete-filled tube with lightweight aggregate achieved the second-highest value, right after concrete with natural aggregate.

When analyzing bridges in Japan, an important advantage of structural concretes based on lightweight aggregates is weight reduction, which is particularly beneficial in seismically active areas. Due to similar structural requirements, this solution can also be effective for bridges located in paraseismic areas. In addition, the use of lightweight concrete can be beneficial under conditions of intense dynamic loading, such as increased rail or tram traffic.

### 5.2. Bridge Structures in Norway

Norway, famous for its trolls and fjords, has also conducted research on the use of lightweight aggregate in bridge constructions [169]. One of the earliest structures of this kind is the Norddalsfjord Bridge, which was built in 1987 [170,171]. This 397 m-long bridge consists of three spans, with the main span measuring 230.5 m. Lightweight concrete using Liapor 8 aggregate was employed for the construction of the main span [172]. As part of the project, strain gauge tests were carried out to determine deformations during both construction and after completion [173,174].

In work [174], the Stolma Bridge was also described, which features the world’s longest box girder span built using the cantilever method [175,176,177]. This bridge, connecting the islands of Bergen and Stavanger, was erected in 1998. For the main span with a length of 301 m, its central portion was constructed using lightweight concrete of class LC60—HF Leca 800 aggregate was used. The reduction in the span’s weight allowed for a decrease in the self-weight, which accounts for about 90% of the total load-carrying capacity of the structure [150]. The remaining part of the bridge was made of normal concrete of class C65, and the foundation was made of concrete of class C45. During the design phase, aspects like the slenderness of the box cross-section, reaching over 14 m in the support area, were analyzed [178,179]. Due to its size and significance, the bridge underwent leveling monitoring [174]. The obtained results indicated that the estimated deflection value was greater than the actual deflection.

**Figure 7:**
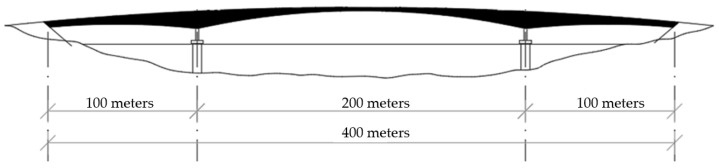
Sketch of the longitudinal section of the Støvset bridge.

The last structure described in work [174] is the Støvset Bridge [180]. This bridge, with a total length of 420 m, was built in 1993 (Figure 7). The main span was partially constructed using lightweight concrete of class LC55. The mixture includes lightweight aggregate Liapor 8, along with natural sand. The detailed composition of the concrete mixture is presented in the study [181]. The remaining elements of the bridge were made from concrete of class C55.

Furthermore, epoxy-coated bars were used as reinforcement due to the bridge’s contact with seawater. Their incorporation into the structure aims to gain experience in using this type of material and to enhance the durability of the structure.

This bridge, like the others, underwent deformation measurements both during and after construction [174]. The measurements of deflections and deformations allow for adjustments in the calculation of concrete strength and the estimation of the modulus of elasticity.

The next cantilever bridge is the Sandhornøya Bridge. It was opened for use in 1989 and is considered one of the oldest structures built using lightweight aggregate. The bridge consists of three spans with a total length of 374 m. In this case, the side spans, with a length of 110 m, were constructed using lightweight concrete of class LC55, while the main span was made from regular concrete of class C45 [75]. Lightweight aggregate Liapor 8 [172] and natural sand were used for production. A detailed description of the mixture’s composition can be found in [182]. Due to its experimental nature, a series of analyses and deformation measurements were conducted [180]. Based on the obtained research results, conclusions were drawn regarding the execution of future structures of this type. For instance, the higher than expected occurrence of cracks was probably a consequence of the improper compaction of the mixture.

Another interesting structure in Norway is the Rugsund Bridge [181]. It connects two islands, the topography of which generates air currents of up to 50 m/s. Initially, the project involved constructing a 3-span cantilever bridge with a box cross-section made of regular concrete of class C55. The total length of the structure is 311 m [22]. However, an alternative concept that proposed using lightweight aggregate for the construction of the main span turned out to be more favorable. By utilizing lightweight concrete of class LC60, the main span was extended by 20 m, in turn reducing the amount of steel required and bringing the pillars closer to the shoreline [183]. Moreover, the amount of ballast used to fill the side spans was also reduced. As a result of these decisions, the total costs decreased by 15%, despite the lightweight aggregate being sourced from the United States [38].

**Figure 8:**
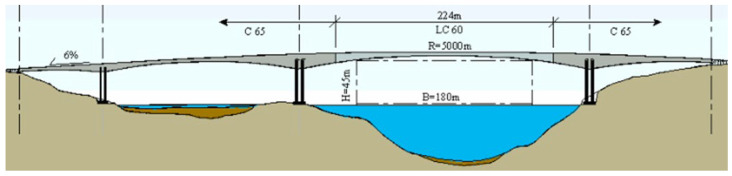
Fragment of the Raftsundet Bridge design documentation [184].

However, the first structure built with the assistance of lightweight volcanic-originated aggregate in the Scandinavian countries was the Raftsundet Bridge [75,184]. This bridge, located in the northern part of Norway, connects two islands in the Lofoten archipelago. The terrain’s characteristics led to the creation of winds in the strait with speeds of up to 60 m/s. Moreover, the area is within a seismic activity zone. These unique topographical conditions prompted designers to consider either a cantilever or a suspension bridge design. Ultimately, a 4-span cantilever bridge with a box cross-section was chosen [179]. Before commencing construction, detailed studies were conducted to define the nature of the wind load prevalent in the area [149]. The main span of the bridge, measuring 298 m in length (Figure 8), was constructed using lightweight concrete of class LC60, while the side spans were made of regular concrete of class C65 [181].

The Nordhordland Bridge illustrates the possibility of using LWA in a different type of bridge structure [152,175,185]. This bridge, completed in 1994, consists of a cable-stayed bridge and a pontoon bridge [1]. The shape of the structure was determined by the depth of the water obstacle and the intense maritime traffic. Lightweight aggregate (LWA) of the Leca type was used to create the bridge deck, resulting in concrete of class LC55. Additionally, 10 pontoons were constructed using Liapor 8 lightweight aggregate, also achieving a concrete class of LC55. A detailed description of the composition of each mixture is provided in study [181]. The pontoons themselves were fabricated in a dry dock and then floated to the construction site. The use of lightweight aggregate contributed to reducing the weight and overall costs of the structure [22].

Another example is the Bergsoysundet floating bridge [1]. This bridge, completed in 1991, is considered the second-longest pontoon bridge in the world. Interestingly, due to the lack of research on detecting the seabed, a decision was made to anchor the ends of the bridge without anchoring the pontoons. This means that the structure is exposed to horizontal tension caused by waves and wind. Due to its unique mode of operation, the structure is continuously monitored [181]. During the analytical model development stage, the designers had to conduct a detailed analysis of the bridge’s dynamic behavior [186]. For this purpose, a modal analysis based on the vibrations of the surroundings (OMA) was performed using frequency measurements of the wind. The analysis was conducted using the Stochastic Subspace Identification (SSI) and Frequency Domain Decomposition (FDD) methods. The results of the analysis indicate the need for further work on refining the models that were used.

The seven pontoons, each with dimensions of 20 m × 24 m and thicknesses of 6.1 and 7 m [182], were constructed using Liapor 8 lightweight aggregate and natural sand. As a result, a mixture with a characteristic compressive strength of 53.9 MPa was obtained. A detailed description of the mixture composition is provided in study [181].

The Boknasundet Cantilever Bridge, completed in 1991, was also lightened using lightweight aggregate [75,187]. The initial design of the structure envisaged the construction of a 5-span bridge with a maximum span length of 150 m. The use of lightweight aggregate allowed the main span to be extended to 190 m and two intermediate piers to be eliminated [179]. This led to savings of approximately 6% of the total project cost. Similarly to the Sandhornøya Bridge, Liapor 8 lightweight aggregate and natural sand were used in its construction. Details regarding the composition of the mixture can be found in the referenced study [181]. Two years after the bridge was put into service, a series of boreholes and sample collections were conducted. Based on the collected samples, the chloride penetration depth was determined. The results of these tests were consistent with the project design assumptions.

As part of a summary of Norwegian bridges made with structural lightweight concrete, Table 9 summarizes the basic information regarding the structures shown.

To summarize the analysis of bridges in Norway, special attention should be paid to the need to relieve the structure due to the specific load bearing system. Due to the shape of the bottom of water obstacles, it is often not possible to construct intermediate supports, forcing the use of cantilever structures or floating bridges. In both cases, the dead weight of the structure is a key factor, so reducing it can significantly improve the efficiency of the structure, especially in conditions of extremely unfavorable terrain.

### 5.3. Bridge Structures in United States of America

Many consider the United States of America to be the pioneer in using lightweight aggregates in concrete applications [188]. According to [29], the use of Haydite lightweight aggregate [26] in bridge construction dates to the 1920s. Currently, the majority of such structures can be found in the states of Virginia and Alaska [189].

One of the earliest documented cases of using lightweight concrete in bridge construction is the eastern section of the San Francisco–Oakland Bay Bridge [190,191]. Built in 1936, it is an example of a double-deck suspension bridge. To reduce the weight of the upper deck, lightweight expanded clay aggregate was used [192,193]. This approach reduced the mass of the structure by 14,300 tons (31.6 million pounds), with estimated savings of 3 million dollars. The effectiveness of this material is confirmed by the 1961 retrofit of the lower deck of the bridge using a similar mixture [22].

The Benicia-Martinez Bridge, opened in 2007, is a structure with a similar design [185]. It consists of 22 spans and was constructed using the cantilever construction method. Due to its location, seismic loads had to be considered [194]. To construct the box girder sections, lightweight aggregate in the form of volcanic rock was used [38], along with natural sand. This resulted in concrete with a density of 125 pounds per cubic foot (approximately 2.002 g/cm^3^) and a compressive strength of 6.5 ksi (about 45 MPa) [22]. Interestingly, due to the high cement content, additional cooling measures were necessary during construction. The concrete’s temperature during curing was controlled by adding ice to the mix instead of water, and a system of pipes circulated liquid nitrogen. Additionally, during the driving of the foundation piles, special bubble curtains were used to dampen vibrations [194].

The I-895 Bridge over the Patapsco River Flats in Baltimore is one example of using lightweight aggregate concrete in a modernization project [22]. This bridge, built in 1957, consists of 42 simply supported spans with a total length of 726 m. Due to the poor condition of the bridge deck and superstructure, a decision was made in 2019 to modernize the structure. The goal of the modernization was to increase the bridge’s capacity without exceeding the load-bearing capacity of the existing piers and piles. The presented repair options favored the use of lightweight concrete for the bridge deck. Using lightweight aggregate in the form of volcanic rock [38] reduced the concrete density to 100 pounds per cubic foot (approximately 1.602 g/cm^3^) [195], while at the same time maintaining a compressive strength of 4.5 ksi (31 MPa).

The Shasta Arch Bridge on Southbound I-5 in Shasta County is another example of using lightweight aggregates to correct design errors [22]. The newly designed arch bridge was intended to replace an existing bridge built in the 1940s. The original project for the new bridge specified the use of regular concrete with a class of C20/25. After constructing the arch, a nonlinear, time-dependent second-order analysis was conducted, which revealed that the foundation’s capacity had been exceeded [158]. This necessitated the use of lighter alternatives for the rest of the structure. As a result, the box girder structure of the bridge, along with its components, was constructed using lightweight concrete with Haydite aggregate [196].

Ozyildirim et al. [197] discuss concrete lightweight aggregate in the context of bridge structures in Virginia. As a leader in Accelerated Bridge Construction (ABC) techniques, they have completed several projects using lightweight aggregate. For example, the Woodrow Wilson Bridge over the Potomac River, which was built in 1962 [198]. Twenty years later, a decision was made to replace the bridge deck, which was originally made of regular concrete, due to rapid wear and tear. Prefabricated deck panels made from expanded shale were chosen for the replacement [38]. This decision was influenced by the possibility of increasing the thickness of the elements, reducing dead loads, and lowering costs [22,197,199]. In 2007, visual and material inspections were conducted on the deck panels, as detailed in a report [200]. Based on this report, damage to the panels near the expansion joints was noted. The concrete properties had slightly improved. At the same time, the authors of the report highlighted the faster replacement capability of these elements during maintenance work.

Meanwhile, the renovation of the Coleman Bridge over the York River in Yorktown [201] proves that working time also matters. Due to the lack of alternative river crossings in the area, the project aimed to keep traffic disruption to less than 12 days [202]. As a result, ready-made segments, with their equipment, were assembled 40 miles from the site and transported to the construction site by barges. The bridge itself was closed for only 9 days [151]. This success was achieved by using lightweight concrete for the deck [199]. It reduced the structure’s weight, matched the capacity of the existing piers, and significantly reduced the time and costs of the renovation [185,203].

A similar approach was taken during the replacement of the I-95 Bridge over the James River [204]. The construction work took place in 2002. The bridge consisted of 5 spans with a total length of approximately 1276 m (4185 feet). To minimize traffic disruptions and adhere to the construction schedule, work was conducted at night. Prefabricated deck panels, combined with a steel superstructure, were transported and installed between the spans [199,203]. Following the successful completion of this project, another 11 bridges on the I-95 route were scheduled for renovation [205,206].

Other bridges that did not require accelerated work were those along Route 33 over the Mattaponi and Pamunkey Rivers. They were meant to replace the Lord Delaware swing bridge, which was built in 1945 [207]. The existing bridge, with a length of 1081 m (3545 feet), had spans of up to about 73 m (240 feet). Due to the satisfactory condition of the existing piers, it was decided to repair them and to then support a new superstructure on them [197]. Because of weak soils and foundations, both the girders and the bridge deck were made of lightweight concrete [22]. Due to the experimental nature of the work, before the bridges were constructed, two full-size girders were cast from the designed mixture at the Turner-Fairbank Highway Research Center of the Federal Highway Administration in McLean and then subjected to testing [208]. Subsequently, both the girders and the bridge deck were cyclically tested, and the results were compared with laboratory data [208,209,210]. Based on these results, the effectiveness of using lightweight concrete in terms of reducing load and costs was confirmed.

In 2001, east of the city of Richmond, the Route 106 Bridge over the Chickahominy River was constructed [211]. This bridge was the first all-lightweight concrete bridge with high strength in Virginia. The structure consists of three spans, each 85 feet long. Each of the spans consists of five precast prestressed concrete stringer beams made of lightweight Stalite aggregate [38], combined with a lightweight concrete deck. One of the spans was additionally strengthened against punching by adding distributed fiber reinforcement. Before the construction work started, a series of tests were conducted to confirm the validity of the assumptions made. Work [212,213] describes the tests of the prestressed concrete stringer beams made of lightweight concrete, and focuses on parameters such as transfer length, dispersion length, flexural strength, and allowable dynamic loads. The results indicate the conservatism of the normative assumptions [214], albeit with significant safety margins in some cases. Meanwhile, the research presented in [215] demonstrates the behavior of the precast stringer beams with a prepared bridge deck. The results confirm the effectiveness of using lightweight high-strength concrete in bridge construction.

The examples mentioned above do not encompass all the lightweight aggregate concrete bridges built in the United States. Additional examples of such projects are presented in Table 10. Based on this table, it is possible to estimate the range of parameters for lightweight concrete for various structural elements. For the bridge deck, mixtures with compressive strengths ranging from 3 to 6 ksi (21 MPa to 41 MPa) and densities from 95 pcf to 120 pcf (1521 kg/m^3^ to 1922 kg/m^3^) are used. For structural elements such as girders and box sections, lightweight concretes with compressive strengths ranging from 3.5 to 9 ksi (24 MPa to 62 MPa) and densities from 103 pcf to 133 pcf (1650 kg/m^3^ to 2130 kg/m^3^) are used.

When analyzing the literature and the number of bridges built using lightweight aggregates, America is the leader in this field. The design guidelines used there form the basis for the development of construction standards in other countries, both in Europe and Asia. The examples of bridges presented emphasize the importance of rapid replacement of individual structural elements and their prefabrication, which helps to shorten investment times and increase the durability and economic efficiency of structures.

### 5.4. Bridge Structures in Europe

**Figure 9:**
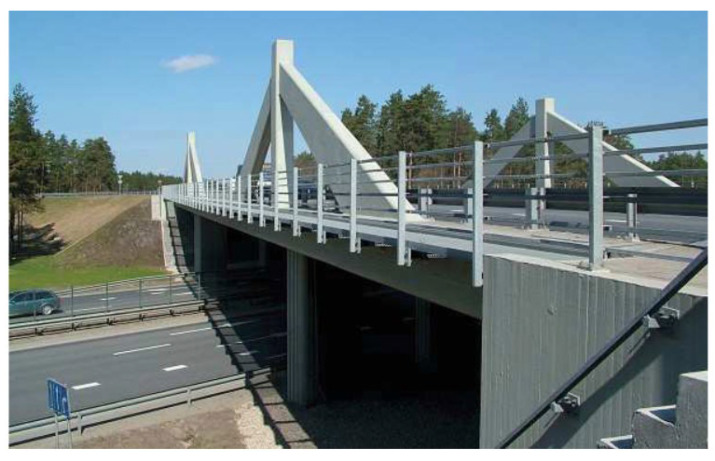
Abava river bridge [221].

It is not only in the USA that lightweight aggregate concrete is used in bridge repairs and construction, but also in Lithuania, as illustrated by examples such as the relief of the stone bridge over the Abava River (Figure 9), the reconstruction of the bridge in the city of Jelgava, and the newly designed viaduct over the A2 highway [216,217,218]. In two of the cases mentioned, the goal of modernization was to increase the capacity of the structures while at the same time reducing their load-bearing demands on the existing piers and soils [219,220]. The last-mentioned structure is an example of designing a construction using lightweight high-strength concrete with a compressive strength of 70 MPa and a density of 1.990 g/cm^3^ [221]. Other examples of constructing bridge structures using lightweight aggregate are provided in work [222]. In work [218], a design for a pedestrian and bicycle viaduct with a composite support system is also presented. It consists of two steel tubes filled with lightweight concrete. Such structures have previously been analyzed in Japan [168].

**Figure 10:**
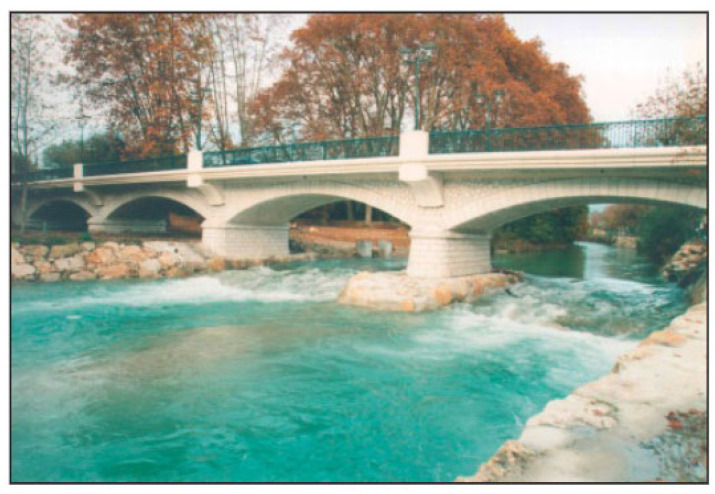
Visualization of the Villeneuve-Loubet bridge repairs [224].

Within Europe, lightweight concrete is also used for the modernization and construction of bridge structures. For example, the Villeneuve-Loubet stone bridge in France (Figure 10) [223]. Due to damage to one of the bridge’s piers and the desire to widen the structure, a decision was made to create a cantilevered sidewalk plate made of lightweight concrete [224].

In the case of the Beaumont-sur-Oise bridge, the design challenge was the deep-seated supportive soil conditions [225]. To address this issue, a decision was made to use lightweight aggregates to reduce the load on the bridge. As part of this approach, a section of the main span measuring 120 m in length was constructed using lightweight concrete [226].

For German bridge structures made of lightweight concrete, you can include the bridge over the Rhine River in Cologne [29]. This structure, with a box girder cross-section and a length of 437 m, was completed in 1979. Its purpose was to alleviate traffic on the adjacent existing steel bridge, which was rebuilt after World War II. The new bridge consisted of five variable-length spans, with part of the main span constructed using lightweight concrete, specifically, LB45 mix. This lightweight concrete included expanded clay aggregate Liapor 8, along with natural sand. The remaining portion of the structure was made using regular concrete with a class of B55. A detailed description of the concrete mixtures is provided in referenced work [227]. Additionally, a series of tests were conducted on the proposed mixture parameters, including compressive strength, modulus of elasticity, shrinkage, and creep. The results confirmed the validity of the chosen design parameters.

The Iroise Bridge over the Elorn River, a cable-stayed bridge, was also constructed using lightweight aggregate [228,229]. This bridge, with a total length of 800 m, and composed of three spans, was completed in 1994. Lightweight concrete, made from expanded shale aggregate and natural sand, was used for constructing the bridge deck of the main span. This decision resulted in reducing the total weight of the structure by 2200 tons [230]. Ultimately, lightweight concrete with an average compressive strength of 45 MPa and a modulus of elasticity of 23 GPa was achieved.

The Friarton Bridge in Scotland, completed in 1978, was originally designed as a 9-span steel structure. The design included a steel box girder with an asphalt surface on an orthotropic steel deck [29]. However, due to changes in geometry, the final decision was made to construct a reinforced concrete bridge deck that was integrated with the upper part of the steel girder. Using lightweight concrete in the project allowed for the retention of the previously designed piers and foundations. Initially, the plan was to develop a mixture with Lytag lightweight aggregate with a density of 1680 kg/m^3^ and an average compressive strength of 38 MPa. Eventually, some concrete with a density of 1690–1750 kg/m^3^ was achieved [112]. One of the construction challenges was related to the workability of the concrete. However, just before placing the mixture, water was added to improve its flowability without significantly affecting its strength.

The Avelengo Bridge, located in the northern part of Italy, also illustrates the potential of using lightweight concrete. This structure is situated at an elevation of 1250 m and spans the Sinigo River canyon [112,231]. Due to the terrain’s topography, a decision was made to construct a single main span with a length of 125 m. The total length of the bridge, including a box section, is 158 m. By using the cantilever construction method for the bridge, attempts were made to balance the loads during construction. Consequently, the box section outside the main span was filled with lightweight concrete, and the bridge deck itself was made from expanded clay aggregate.

In study [232], a numerical reliability analysis of lightweight reinforced and prestressed concrete structures was conducted using Monte Carlo simulations, with the Avelengo bridge chosen as the case study. The selection of this structure was based on its strategic importance, structural configuration, and environmental conditions. The results obtained confirmed the effectiveness of the proposed nonlinear numerical analyses.

Another interesting example is the Koningspleijbrug Bridge in the Netherlands, completed in 1985 [233]. The bridge consists of 13 spans (12 approach spans and 1 over the river), with the lengths of the spans as follows: 37 + 4 × 49 + 80.54 + 133.43 + 80.54 + 4 × 49 + 37 m. Box sections were used over the obstacle and adjacent spans, while the remaining spans featured twin-web sections. During the design phase, a decision was made to use lightweight concrete for financial and environmental reasons [112]. Interestingly, this material was also used for the bridge’s piers and abutments. Lytag pozzolan lightweight aggregate obtained from the Lytag VASIM factory near the Nijmegen power plant was used as the lightweight aggregate. As a result, the total mass of the structure was reduced by 20%, with compressive strength estimated at 40–50 MPa. The authors also highlight the insulating properties of the lightweight aggregate, which can be problematic when constructing large-scale structures.

The first recorded use of lightweight aggregate in bridge construction in Poland dates back to the 1970s [234]. Comparative studies were conducted during this research by creating full-scale models of a bridge using lightweight aggregate in the form of sintered shale and natural aggregate. As a result, lightweight concrete with compressive strengths of up to 45 MPa for prefabricated beams and up to 30 MPa for the bridge deck was achieved [29].

However, the use of lightweight concrete in bridge structures is rare in Poland. There are known cases where it has been used for additional elements such as sidewalk curbs or railings. For instance, in the construction of a road bridge in Torun [235], lightweight concrete with Pollytag lightweight aggregate made from fly ash was used for the construction of sidewalk curbs and technical shoulders. The authors of the study paid significant attention to describing the design procedure and the mixing of the concrete mixture.

Lightweight aggregate concrete was also used for the construction of the viaduct over the market square in Chorzow [179,234]. However, due to design and construction errors, it was reconstructed and strengthened in 2013 [236,237].

A breakthrough came with the research projects COMBRIDGE and FOBRIDGE [238]. Thanks to these projects, the first composite road bridges and the first composite pedestrian footbridges were built in Poland. The first fully composite bridge is a single-span bridge built in the town of Nowa Wieś near Rzeszow over the Czarna stream in 2016. Lightweight concrete based on ash aggregate was used in its construction. A mixture of class LC 30/33 was used to make the sidewalk caps. Dynamic tests of the bridge were also conducted shortly after its completion [157].

The first composite-concrete bridge in Poland is a structure built in 2015 in the town of Blazowa [239,240]. Its main structure consists of four composite girders combined with a concrete bridge deck using lightweight aggregate concrete. The girders were additionally reinforced with two concrete crossbeams of the same composition as the deck. The concrete was made from lightweight aggregate and low-alkali cement. Before the actual testing, a series of laboratory tests were conducted on the proposed bridge deck and the girder itself [155,241,242].

When analyzing the use cases of lightweight aggregate in bridges across Europe, there is little interest in this solution. This may be due to the lack of complex terrain obstacles and the lower seismic risk compared to other regions of the world. However, this does not alter the fact that structures using lightweight aggregates are being built and are performing their structural functions. In the context of changes in European Union climate policy, restrictions on the extraction of natural resources and the growing importance of a circular economy, man-made lightweight aggregates are becoming an increasingly attractive alternative. Fly ash aggregates are particularly promising and could play a key role in the further development of lightweight concrete. As a result, it is predicted that interest in this solution will continue to grow.

### 5.5. Use of Lightweight Aggregates for Tunnel Construction

The use of lightweight aggregates for constructing bridge structures is not limited to just bridges. Several studies also consider the use of lightweight aggregates for constructing tunnels, which are categorized as bridge structures.

Study [243] compares the durability of tunnels and their maintenance frequency concerning the materials they are made from. The purpose of this study is to gather existing design experience and research in order to identify future solutions for Norwegian tunnels. The study mentions segments made from lightweight aggregate concrete, emphasizing its relatively low cost, better properties regarding soil pressure/suction, and fire safety. However, due to problems with seepage and the need for frequent maintenance, this solution was rejected.

This does not mean that lightweight concrete is not used in tunnel construction at all. Due to its permeability, it can serve as a protective layer for geotextiles. In study [244], an innovative method of protecting a tunnel’s drainage system using lightweight mortar is presented. Special mortar, along with a foaming solution, enhances drainage by increasing the drainage space, preventing clogging of geotextiles by fine fractions, and improving the drainage system.

The use of lightweight concrete as a fire protection measure is also considered. For example, a numerical analysis presented in study [245] considers the effectiveness of replacing the fire protection layer with a layer made of lightweight concrete. Lightweight concrete’s cost-effectiveness and ecological advantages when compared to conventional protective layers are evident. The analysis, conducted in the ANASYS program, confirms the protective properties of lightweight concrete. This solution’s effectiveness is also confirmed by the analysis in [246].

There is also a consideration for using lightweight aggregates in the construction of floating tunnels. This innovative solution would allow the crossing of deep obstacles while maintaining the navigability of waterways [247]. Due to the unconventional nature of the entire structure, numerous institutes are working on its design procedure [248,249]. Currently, floating tunnels are being considered for construction in Norway and China [250]. Lightweight aggregates, as in the case of floating bridges, would be used in constructing floating pontoons. However, at present, this solution is in the conceptual stage.

An alternative solution, building on the concepts described earlier, is the combination of a floating bridge with a tunnel [251]. This concept uses pontoons and special mooring lines installed along the shore to stabilize the structure. A brief dynamic analysis confirms the effectiveness of the proposed structural solutions. Like floating bridges, lightweight aggregates would be used for the construction of concrete pontoons in such structures.

## 6. Summary and Conclusions of the Literature Review

To summarize the conducted literature review, it can be concluded that lightweight aggregate concretes can be successfully utilized as a construction material in bridge structures. This is supported by the literature review, numerous scientific publications, design guidelines, and examples of bridge structures built using lightweight concretes. The use of lightweight aggregates in bridge construction dates back to the early 1960s, indicating that the design of bridge structures with lightweight aggregates has been around for over 50 years.

Currently, based on decades of experience, designers have developed a set of design guidelines regarding the use of lightweight aggregates in bridge structures. Utilizing these guidelines, new bridge structures are designed using lightweight aggregate concrete (LWC). Moreover, design concepts for underwater floating tunnels are evidence of the further potential development of lightweight concretes in bridge engineering.

Furthermore, the literature analysis indicates an increasing interest in bridge structures made of lightweight aggregates over the past 10 years. Based on this, there has been a rise in research analyzing the ecological aspects of lightweight aggregates and their resistance to environmental factors. This indicates the necessity for further research on LWC.

The conclusions from further research on the use of lightweight aggregates in bridge engineering, considering the resistance of such aggregates to various environmental impacts, will contribute to the development of currently available design guidelines, and increase awareness among designers.

## 7. Future Research and Perspectives

The purpose of this study was to conduct an analysis of the current state of knowledge regarding the use of lightweight aggregate concrete in bridge construction. Based on the results of the analysis, fundamental assumptions were defined regarding the use of lightweight aggregates in bridge engineering. Existing design guidelines for bridge structures were compiled, and potential pathways for further development of the analysis of lightweight aggregate concrete structures in bridge construction were outlined.

The descriptions of bridge projects presented in Section 5, and the guidelines for the use of lightweight aggregates in structural concrete outlined in Section 4 provide an overview of the limited types of aggregates used in the production of structural concrete. Therefore, further research will focus on analyzing the potential use of other lightweight aggregates in bridge construction, with particular emphasis on aggregates from recycling and aggregates derived from waste processing. Subsequently, attempts will be made to produce lightweight aggregate concrete with the addition of waste materials from PET bottle recycling. The research will involve developing the composition of lightweight aggregate concrete with the addition of waste materials, establishing an optimal firing curve, and conducting a series of tests to verify the mechanical and chemical properties of the newly obtained aggregate.

The aim of these efforts is to streamline the waste processing process, especially for materials currently considered non-recyclable.

## Figures and Tables

**Table 1:** Summary of keyword occurrences for each database (accessed on 30 January 2025).

Selected Keywords	Number of Phrases		
	(Scopus)	(LENS)	(WorldCat)
**Step 1**			
concrete	588,385	979,020	1,317,812
bridge	540,728	1,070,255	1,010,362
aggregate	456,831	873,820	598,455
lightweight	176,382	194,123	356,571
**Step 2**			
concrete + bridge	51,815	61,906	14,442
concrete + aggregate	55,453	69,796	22,776
concrete + lightweight	13,183	15,862	8751
lightweight + aggregate	9067	11,355	5515
bridge + aggregate	5002	10,488	555
bridge + lightweight	3244	3587	778
**Step 3**			
concrete + aggregate + lightweight	6361	7449	2427
concrete + bridge + aggregate	1658	1999	195
concrete + bridge + lightweight	1040	1047	204
bridge + aggregate + lightweight	314	379	63
**Step 4**			
concrete + bridge + aggregate + lightweight	265	294	0

**Table 6:** Description of the composition and parameters of the lightweight structural concretes used for bridge engineering [128].

Component	Proportion 6 ksi (41.37 MPa)	Proportion 8 ksi (55.16 MPa)
Cement CEM III	504 Ib (228.61 kg)	671 Ib (304.36 kg)
Fly ash class C	168 Ib (76.20 kg)	316 Ib (143.34 kg)
Lightweight aggregate expanded clay	1264 Ib (573.34 kg)	1123 Ib (509.38 kg)
Sand	1149 Ib (521.18 kg)	1029 Ib (466.75 kg)
Water	222 Ib (100 kg)	247 Ib (112.04 kg)
Compression strength after 1 day	4000 psi (27.57 MPa)	5500 psi (37.92 MPa)
Compression strength after 28 day	7200 psi (49.64 MPa) (laboratory) 7800 psi (53.78 MPa) (in field)	8600 psi (59.29 MPa) (laboratory) 7500 psi (51.71 MPa) (in field)
Density	118 pcf (1890 kg/m^3^)	122 pcf (1954 kg/m^3^)

**Table 7:** Classification of lightweight concrete based on density [18].

Density Class	D1.0	D1.2	D1.4	D1.6	D1.8	D2.0
**Density range [kg/m^3^]**	≥800 ≤1000	≥1000 ≤1200	≥1200 ≤1400	≥1400 ≤1600	≥1600 ≤1800	≥1800 ≤2000

**Table 8:** Comparison of European norm requirements for designing concrete in bridge structures [126,138].

Requirements for Concrete in Bridge Structure Construction	Applicable Conditions
Cement Class:	CEM I
Compressive strength class [MPa]	From C20/25 (from C30/37 for newly designed structures)
Exposure class:	XA, XC, XF, XS, XD
Consistency class	S2–S4
Water	V1–V3
Maximum aggregate grain size class (Dmax):	Dmax = 16 mm
Aggregate absorption:	Max 4%:
Frost resistance	F150

**Table 9:** Overview of Norwegian lightweight concrete structures.

Lp.	Name	Year of Completion	Type of Lightweight Aggregate	Bridge Components	Density [kg/m^3^]	Cube Strength [MPa]
1	Raftsundet	1998	Stalite + Natural sand	Full	1950	60
2	Sandhornøya	1989	Liapor + Natural sand	Deck + Beams	1950	55
3	Støvset	1993	Liapor + Natural sand	Full	1924	55
4	Stolma	1998	LECA + Natural sand	Full	1950	60
5	Norddalsfjord	1987	Liapor + Natural sand	Full	N/A	45/35
6	Nordhordaland	1994	LECA + Natural sand	Deck	1900	55
			Liapor + Natural sand	Pontoons		
7	Rugsung	2000	Stalite + Natural sand	Full	1950	60
8	Boknasundet	1990	Liapor + Natural sand	Deck + Beams	1950	60
9	Bergsoysundet	1992	Liapor + Natural sand	Pontoons	1900	53.6
10	Grenland	1996	Liapor + Natural sand	Deck + Beams	N/A	55
11	Eidsvoll	1992	Liapor + Natural sand	Superstructure	1880	55
12	Sunday	2003	Stalite + Natural sand	Full	1970	60

**Table 10:** Summary of structures built in the United States using lightweight concrete.

No.	Name	Country	Year of Completion	Type of Lightweight Aggregate	Bridge Components	Density [kg/m^3^]
1	Coronado Bridge, CA	USA	1969	Expanded shale + Natural sand	Full	1842
2	Antioch Bridge, CA	USA	1978	Baypor F-43 + Natural sand	Full	1842
3	Arthur Ravenel Jr. Bridge (Cooper River Bridge),SC	USA	1992	Solite/Stalite + Natural Sand	Full	1842
4	Brooklyn Bridge, NY	USA	1999	Solite + Natural sand	Deck	1890
5	Neuse River Bridge, NC	USA	1999	Stalite + Natural sand	Full	1842
6	I-95 Bridge over James River, Va	USA	2002	Solite + Natural sand	Deck + Superstructure	1842
7	I-95 Bridges North of Downtown, Va	USA	2014	Solite + Natural sand	Deck	1842
8	Benicia-Martinez Bridge, CA	USA	2007	Stalite + Natural sand	Full	2002
9	Skagit River Bridge, WA	USA	2013	Stalite + Natural sand	Emergency repair	2130/1954
10	San Francisco-Oakland Bay Bridge	USA	1961	Ceramsite + Natural sand	Deck	1522
11	I-895 Bridge over the Patapsco River Flats—Baltimore, MD	USA	2019	Stalite + Natural sand	Deck + Superstructure	1602
12	Shasta Arch Bridge on Southbound I-5—Shasta County, CA	USA	2018	Ceramsite + Natural sand	Box grider	1922
13	Route 198 (Dutton Road) Bridge over Harper Creek—Gloucester County, VA	USA	2016	Stalite + Natural sand	Grider + Deck	1842
14	Woodrow Wilson Bridge over the Potomac River—Washington, D.C.	USA	1983	Stalite + Natural sand	Remove Deck	1842
15	Coleman Bridge over the York River—Yorktown, VA	USA	1983	Stalite + Natural sand	Deck	1842
16	Beach Bridge—Norht Haven, ME	USA	2013	Stalite + Natural sand	Griders	1922
17	Route 33 Bridges over the Mattaponi and Pamunkey Rivers—West Point, VA	USA	2006/2007	Stalite + Natural sand	Grider/Deck	2002/1922
18	Silver Creek Overpass Bridge Utah	USA	1968	Expanded shale + Natural sand	Deck	1762
19	Heart of America Bridge, Missouri	USA	1985	Buildex + Kaw river sand	Deck	1762
20	Ohio Turnpike Twin Bridges, Ohio	USA	1984	Haydite + Natural sand	Deck	1858
21	Sebastian Inlet Bridge, Florida	USA	1964	Solite + Natural sand	Full	1842
22	William Preston Lane Jr. Bridge, Maryland (East boud)	USA	1952	Solite + Natural sand	Full	1650
23	William Preston Lane Jr. Bridge, Maryland (East boud)	USA	1988	Solite + Natural sand	Remove Deck	1682
24	Sebastian Inlet Bridge, Florida	USA	1964	Solite + Natural sand	Full	1842
25	Wabash River Bridge, Indiana	USA	1994	Expanded shale	Griders	2002
26	Pulaski Skyway, NJ	USA	2018	N/A	Deck	1922
27	Ben Sawyer Bridge—Sullivan’s Island, SC	USA	2010	N/A	Deck	1842
28	Massaponax Church Road (Route 608) Bridge over Interstate 95—Spotsylvania County, VA	USA	2009	N/A	Deck	1922
29	Marc Basnight Bridge over the Oregon Inlet—Outer Banks, NC	USA	2019	N/A	Deck	1922
30	Francis Scott Key Bridge—Baltimore, MD	USA	1977	N/A	Deck	1794
31	Route 22 Bridge over the Kentucky River—Gratz, KY	USA	2010	N/A	Griders	2002
32	I-85 Ramp over State Route 34—Newnan, GA	USA	2010	N/A	Griders	1922
33	Boulevard (Route 161) Bridge across the James River—Richmond, VA	USA	1959	N/A	Deck	1762
34	Walt Whitman Bridge Philadelphia, PA	USA	2007	Solite + Natural sand	Remove Deck	N/A
35	William Preston Lane Jr. Bridge, Maryland (West bound)	USA	1975	Norlite + Natural sand	FULL	N/A
36	Parrotts Ferry Bridge, CA	USA	1979	N/A	Full	N/A
37	Sam White Bridge, UT	USA	2011	N/A	Full	N/A
38	Thaddeus Kosciusko Bridge (I-87), NY	USA	2013	N/A	Deck	N/A
39	I-40 Bridge over the French Broad River, TN	USA	2015	N/A	Full	N/A
40	US 15/29 Bridge over Broad Run near Gainesville, VA	USA	2007	N/A	Deck	N/A

## Data Availability

No new data were created or analyzed in this study. Data sharing is not applicable to this article.
